# Action-at-a-distance mutations by 8-oxo-7,8-dihydroguanine: adenine pair triggered by MUTYH

**DOI:** 10.1186/s41021-025-00340-0

**Published:** 2025-10-16

**Authors:** Ruriko Fukushima, Tetsuya Suzuki, Hiroyuki Kamiya

**Affiliations:** Graduate School of Biomedical and Health Sciences, Hiroshima University, 1-2-3 Kasumi, Minami-ku, Hiroshima, 734-8553 Japan

**Keywords:** 8-oxo-7,8-dihydroguanine, 8-hydroxyguanine, Action-at-a-distance mutation, MUTYH

## Abstract

**Background:**

8-Oxo-7,8-dihydroguanine (8-hydroxyguanine, G^O^) is a major damaged base caused by oxidation. Misincorporation of dATP opposite G^O^ by DNA polymerases leads to a G:C→T:A transversion at the damaged site via G^O^:A intermediate formation. The G^O^:A pair is also formed by 8-oxo-7,8-dihydro-2'-deoxyguanosine 5'-triphosphate incorporation opposite A. The G^O^:C and G^O^:A pairs are both repaired through the base excision repair (BER) pathway to suppress the G:C→T:A mutations. G^O^:C also induces action-at-a-distance mutations around the damaged base. These untargeted mutations seem to be induced through the excision of G^O^ from G^O^:C by DNA glycosylases, such as OGG1 and NEIL1, in the BER pathway. The adenine base of G^O^:A is excised by a specific adenine DNA glycosylase, MUTYH, and this excision potentially induces action-at-a-distance mutations.

**Results:**

In this study, plasmid DNA bearing a G^O^:A pair was introduced into human U2OS cells to investigate the untargeted mutations by the G^O^:A pair. The G^O^:A pair induced action-at-a-distance mutations at C bases in 5'-TpC-3' of the G^O^-strand, in contrast to those by G^O^:C, which elicit mutations at G bases of 5'-GpA-3'. Furthermore, the untargeted mutations were suppressed by the MUTYH knockdown.

**Conclusion:**

The G^O^:A pair induced the action-at-a-distance mutations through base excision by the MUTYH glycosylase.

**Supplementary Information:**

The online version contains supplementary material available at 10.1186/s41021-025-00340-0.

## Introduction

Endogenous and exogenous factors constitutively damage DNA, and the damaged bases induce mutations by exacerbating nucleotide misincorporation by DNA polymerases (pols). The DNA precursors are also the targets of various mutagens, and the modified nucleotides are occasionally incorporated opposite incorrect bases in DNA. The accumulation of mutations can lead to carcinogenesis and aging [1, 2]. Living organisms possess various DNA repair mechanisms to suppress mutagenesis and maintain genetic information [3, 4].

8-Oxo-7,8-dihydroguanine (8-hydroxyguanine, G^O^) is a representative type of DNA damage produced by reactive oxygen species (ROS) [5, 6], and forms base pairs with both A and C [7, 8]. Thus, G^O^ in DNA, generated by the oxidation of the G base in DNA and the incorporation of 8-oxo-7,8-dihydro-2'-deoxyguanosine 5'-triphosphate (dG^O^TP) opposite C, causes a G:C→T:A mutation at the damaged site [9–12]. The G^O^-induced mutations have been proposed to be linked to the ROS-associated mutational signature, single-base substitution 18 (SBS18) [13–15]. Typically, G^O^ bases in DNA are managed by base excision repair (BER) to prevent mutagenesis. This system requires two specific DNA glycosylases, OGG1 and MUTYH [16]. The first acting enzyme is OGG1, which excises the G^O^ base in the G^O^:C base pair [17–23]. The second acting enzyme, MUTYH, removes the undamaged A base instead of the G^O^ base in the G^O^:A base pair, which is formed during replication when the G^O^ base has escaped from OGG1 [24–31]. This process provides another chance for OGG1 to excise the G^O^ base after reforming the G^O^:C base pair by dCTP incorporation during the gap-filling.

Human MUTYH is an adenine DNA glycosylase and an ortholog of *Escherichia coli* MutY [24–31]. The substrates of MUTYH are reportedly the A base paired with G^O^ or G. Unlike bifunctional OGG1, which has weak lyase activity in addition to glycosylase activity, MUTYH is a monofunctional glycosylase [27]. NEIL1 and NEIL2 are considered as potential backup enzymes for OGG1 [32], but no backup enzyme has been identified for MUTYH.

The G^O^ base also induces action-at-a-distance mutations; *i.e*., base substitutions at G bases distant from the G^O^ position in human cells [33–36]. These mutations are found at the G bases in 5'-GpA-3' contexts in the strand corresponding to the G^O^-strand of the original DNA, and predominantly in the region upstream of G^O^. The 5'-GpA-3' dinucleotides are complementary to the 5'-TpC-3' dinucleotides, which are the preferred substrates for APOBEC3-family cytosine deaminases [37]. The mutations at 5'-*G*pA-3' suggest that APOBEC3s are associated with the action-at-a-distance mutations. APOBEC3s are believed to be endogenous mutagens and involved in the APOBEC mutational signatures, SBS2 and SBS13 [13, 14]. Among the seven human APOBEC3 family members, the expression levels of APOBEC3A and APOBEC3B are particularly correlated with carcinogenesis [38–40]. Six out of the seven APOBEC3s preferentially deaminate the C bases in the 5'-TpC-3' sequences of single-stranded (ss) DNA to U bases (APOBEC3G prefers the second C base in the 5'-CpC-3' contexts). Indeed, the knockdown of APOBEC3B in human U2OS cells reduced the action-at-a-distance mutations induced by G^O^:C [41]. In this mutagenesis process, DNA pol(s) could incorporate dATP opposite the U bases formed by APOBEC3B, resulting in G:C→A:T mutations. In addition, the U bases would be excised by uracil DNA glycosylase (UNG2) to generate abasic sites, thus inducing various mutations by DNA synthesis across the abasic sites [42]. Our recent study proved that UNG2 is involved in the action-at-a-distance mutations induced by G^O^:C [43]. Additionally, the mutations by G^O^ were decreased by the OGG1 knockdown [44]. These results suggest that the APOBEC3-involved action-at-a-distance mutations are linked to the OGG1-initiated BER that suppresses G:C→T:A mutations by G^O^ (*OGG1 paradox*). The NEIL1 knockdown also reduced these mutations, and the double knockdown of OGG1 and NEIL1 exhibited an additive effect on the mutations [45].

Thus, G^O^ is a double-sided mutagenic base and may be related to SBS2 and SBS13, as well as SBS18. When left within DNA, G^O^ induces G:C→T:A transversions at the lesion site. This base substitution is classified as SBS18. In contrast, the action-at-a-distance mutations occur when G^O^ is removed, and the base substitutions can be assessed as SBS2 or SBS13. It is possible that the G^O^ system suppressing the G:C→T:A transversion is associated with the APOBEC signature.

**Fig. 1:**
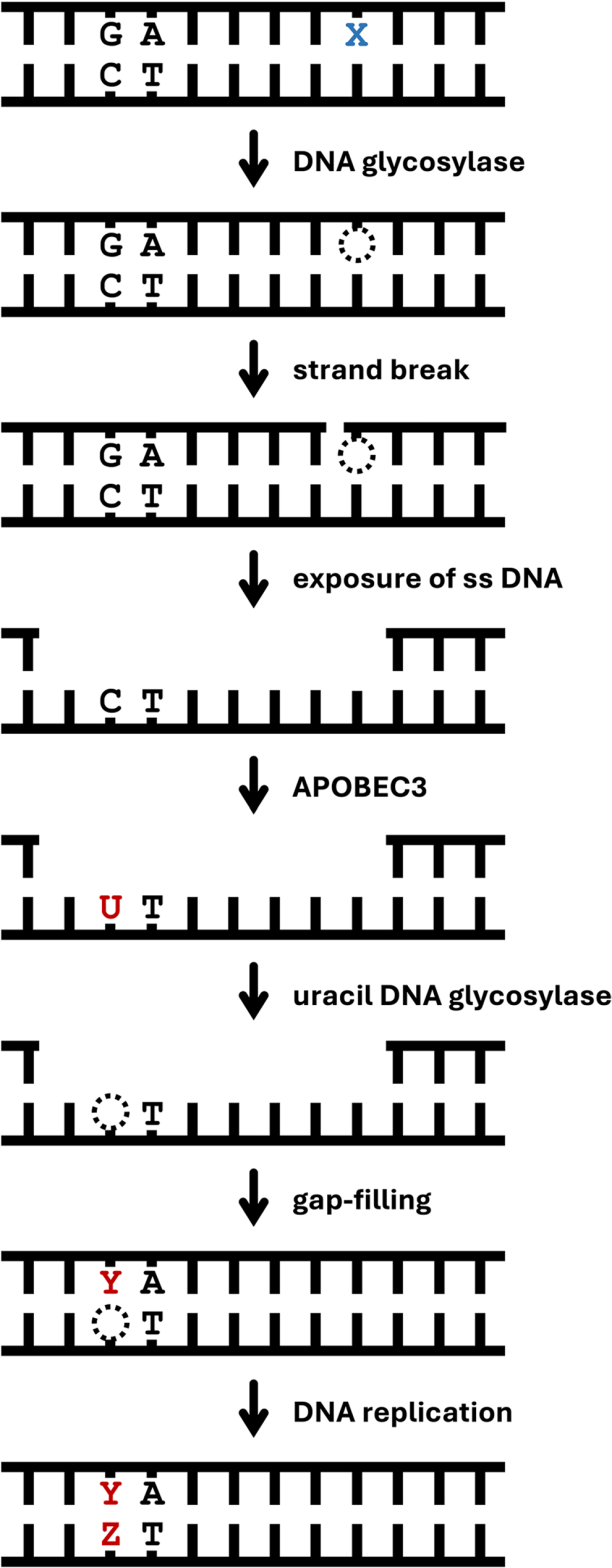
Mutagenesis model through base excision by DNA glycosylase, based on the proposal by Chen et al. [46] and the experimental results [41, 43–45]. An X and a dashed circle indicate a glycosylase-targeted base (*U*:G, *T*:G, *G*^*O*^:C, and *A*:G^O^) and an abasic site, respectively. A mutated base pair is shown as Y:Z

U:G/T:G mispairs induce mutations similar to the action-at-a-distance mutations by G^O^ [46]. In the mutagenesis model proposed by Chen et al. (Fig. 1), the BER intermediates generated after the removal of U or T by DNA glycosylases may be taken over by mismatch repair proteins, leading to the exposure of the strand complementary to the original U/T-containing strand. In the exposed ss DNA, the C bases in the 5'-TpC-3' sequences are deaminated by APOBEC3s, resulting in the mutations at G bases in the 5'-GpA-3' sequences of the U/T-strand. However, Chen et al. did not investigate whether the DNA glycosylases are involved in the mutation process.

DNA pols incorporate dG^O^TP opposite A in addition to C in the template DNA, and the incorrect incorporation opposite A causes A:T→C:G transversions in living cells [47, 48]. In this case, MUTYH promotes the mutations since the protein excises the template A base [49]. We noticed that the A removal by MUTYH possibly induces the action-at-a-distance mutations. The untargeted mutations by the G^O^:A pair were expected to be induced upstream of the A base (downstream of the G^O^ base) since MUTYH removes the undamaged A base, but not the G^O^ base. Moreover, the mutations at C bases in the 5'-TpC-3' sequences of the G^O^-strand would increase. This expected consequence is in contrast to the untargeted mutations induced by G^O^:C; *i.e*., the mutations at G bases in the 5'-GpA-3' sequences in the upstream region of the G^O^-strand.

In this study, we prepared shuttle plasmid DNA carrying a G^O^:A pair and analyzed the action-at-a-distance mutations in human U2OS cells. Additionally, we examined the effect of the MUTYH knockdown on the mutations, given that the enzyme initiates the mutation process. The action-at-a-distance mutations at the 5'-Tp*C*-3' sequences in the G^O^-strand were induced by G^O^:A, and the MUTYH knockdown reduced the mutations. Our present findings, together with the previous reports on action-at-a-distance mutations, suggest a common mechanism for the untargeted mutations initiated by the DNA glycosylase, referred to as the *DNA glycosylase paradox*.

## Materials and methods

### Materials

5'-Phosphorylated oligodeoxyribonucleotides (ODNs) containing G or G^O^ for plasmid DNA construction were synthesized and purified by HPLC, as described previously (Table 1) [49]. The Stealth siRNA against MUTYH (si-MUTYH) and the Stealth RNAi Negative Control Lo GC duplex (%GC 36) were purchased from Thermo Fisher Scientific (Waltham, MA, USA). The other ODNs were purchased from Integrated DNA Technologies (Coralville, IA, USA) and Fasmac (Atsugi, Japan). The human osteosarcoma U2OS cells were obtained from the American Type Culture Collection (Manassas, VA, USA, ATCC HTB-96).

**Table 1:** Oligonucleotides used in this study

Oligonucleotide	Sequence (5' → 3')^a^
ODNs for plasmid construction	
ODN-1^a^	P-dGATCCGGCGGCGCAGCACCAT
ODN-2^a^	P-dGATCCGGCGG^O^CGCAGCACCAT
ODNs for PCR-RFLP	
supF-SV40L(KpnI) Fw	dGGTTCTTTCCGCCTCAGAAG
supF_L336	dGAAGCCAGTTACCTTCGGAA
siRNA	
si-MUTYH sense	UCACAUCAAGCUGACAUAUCAAGUA
si-MUTYH antisense	UACUUGAUAUGUCAGCUUGAUGUGA

^a^P represents the phosphate

### Plasmid DNA construction

**Fig. 2:**
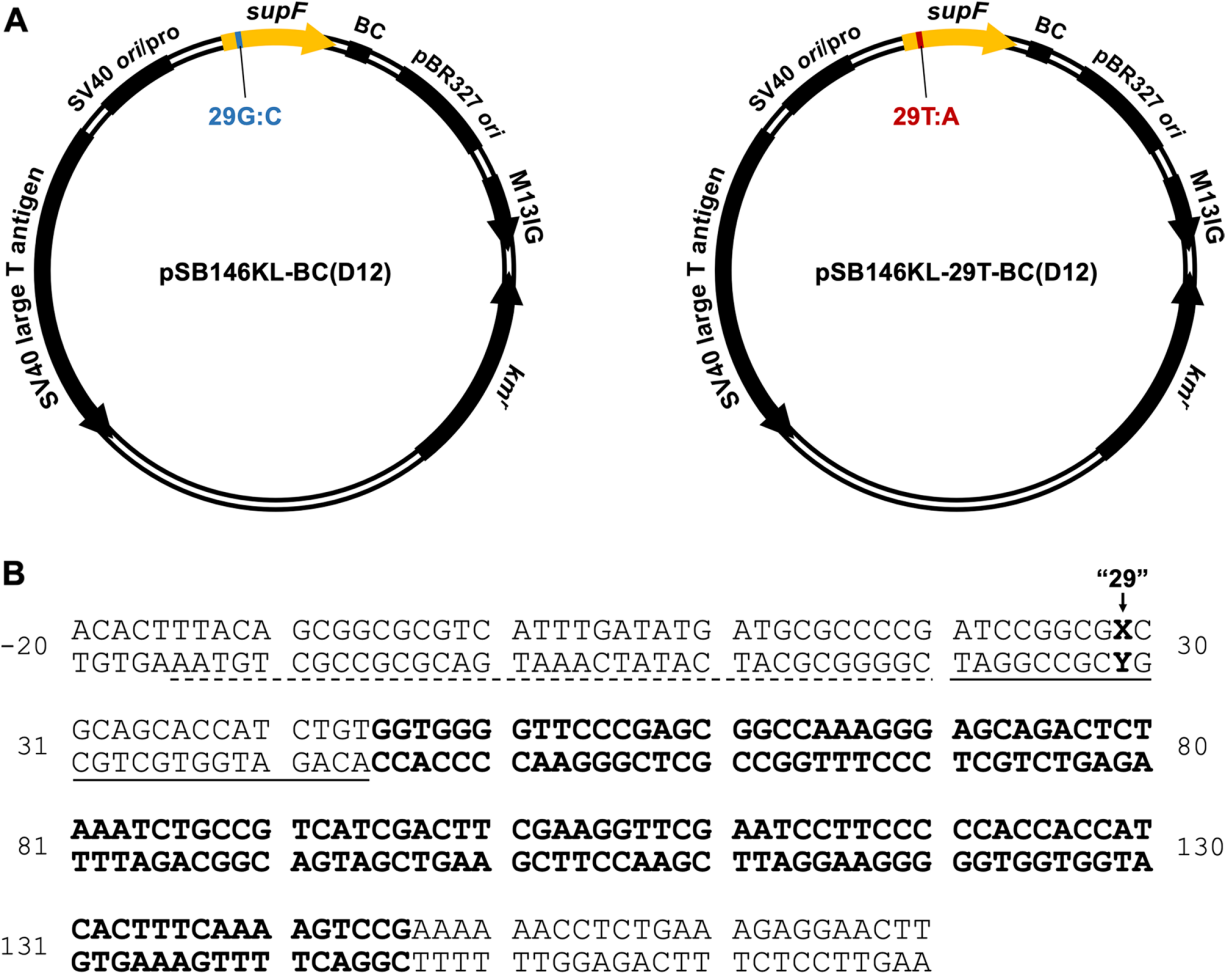
Plasmids used in this study. **A** pSB146KL-BC12(D12) and pSB146KL-29T-BC(D12) containing G:C and T:A, respectively, at position 29 of the modified *supF* gene. BC, barcode sequence (N12, N = A, T, G, or C); *ori*, replication origin; pro, promoter; SV40 large T antigen, SV40 large T antigen (E107K/D402E) gene; M13IG, intergenic region of M13 phage DNA; *km*^*r*^, kanamycin resistance gene. **B** Nucleotide sequence of the modified *supF* gene (bold letters) including its upstream and downstream sequences in pSB146KL-BC(D12)/pSB146KL-29T-BC(D12). Dashed and solid underlines represent the promoter and the pre-tRNA sequences of the gene, respectively. Position 29 is shown as X:Y

The base pair at position 29 of the *supF* gene on the plasmid pSB146KL-BsmBI [36] was changed from G:C to T:A, using a QuikChange Lightning Site-Directed Mutagenesis Kit (Agilent Technologies, Santa Clara, CA, USA) and the primer set supF176T S (5'-dAGATCCGGCGTCGCAGCACCA-3') and supF176T AS (5'-dTGGTGCTGCGACGCCGGATCT-3'), to generate pSB146KL-29T-BsmBI. Barcode sequences (random 12-nucleotide sequences) were inserted into pSB146KL-BsmBI and pSB146KL-29T-BsmBI, as described previously [36]. The resultant plasmids were named pSB146KL-BC(D12) and pSB146KL-29T-BC(D12), respectively (Fig. 2A). The double-stranded (ds) pSB146KL-BC(D12) plasmid with a G:C base pair and the ds pSB146KL-29T-BC(D12) plasmid with a G:A or G^O^:A base pair at position 29 (Fig. 2B) were constructed using the ss pSB146KL-BC(D12) or pSB146KL-29T-BC(D12) plasmid and 5'-phosphorylated G or G^O^ ODN (Table 1), as described previously [36, 50].

### siRNA and plasmid DNA transfections and supF mutation analyses

U2OS cells (2.5 × 10^5^ cells/well in 6-well plates) were reverse transfected with 0.625 µL Lipofectamine RNAiMAX (Thermo Fisher Scientific) and 7.5 pmol siRNA (final concentration 2.5 nM). The Stealth RNAi Negative Control Lo GC duplex was used as the negative control RNA. At 24 h after siRNA treatment, 500 ng (148 fmol) of the plasmid DNA was transfected with Lipofectamine 2000, as described previously [36]. The plasmid DNA was extracted from the cells after 48 h incubation and treated with *Dpn*I to digest unreplicated plasmids, as described previously [33]. To calculate the *supF* mutant frequency, the replicated plasmid was electroporated into the *E. coli* RF01 strain [51]. The *supF* gene of the mutant plasmids was sequenced to analyze the mutation spectra. The substitution frequencies at the C bases of 5'-TpC-3' (*F*_TpC_) were calculated by multiplying the *supF* mutant frequencies by the proportion of mutants with base substitution(s) and the number of base substitutions at 5'-Tp*C-*3' per plasmid DNA molecule containing base substitution(s), as the substitution frequencies at the G bases of 5'-GpA-3' [35].

### Measurement of G/T ratio at position 29

The region around position 29 of the *supF* gene (201 bp) was amplified by PCR, using the extracted plasmid as the template and supF-SV40L(KpnI) Fw and supF_L336 as the primers (Table 1). Mixtures with certain ratios of pSB146KL-BC(D12) and pSB146KL-29T-BC(D12) extracted from *E. coli* were also used as templates, to generate a calibration curve. The PCR products were treated with *Bsa*HI for 3 h and analyzed by 3% agarose gel electrophoresis. The band intensities were quantified using the ImageJ software [52] after staining with GelRed Nucleic Acid Gel Stain (Biotium, Fremont, CA, USA), and the ratio of the plasmids with G:C at position 29 to those with T:A was calculated from the calibration curve.

Sanger sequencing of the PCR products was performed, and each electropherogram datum was analyzed for the base at position 29 with EditR [53]. The ratio of the plasmids with G:C at position 29 and those with T:A at position 29 was calculated from the respective calibration curves for G:C and T:A.

### Western blotting

Whole cell extracts were prepared at 24, 48, and 72 h after siRNA introduction. The extracts were fractionated by SDS-10% polyacrylamide gel electrophoresis. After protein transfer to PVDF membranes, the membranes were blocked with 3% skimmed milk in phosphate-buffered saline containing 0.05% Tween 20 (PBS-T) for 1 h at room temperature. Rabbit anti-MUTYH (Proteintech, Rosemont, IL, USA, catalog No. 19650-1-AP) and mouse anti-β-tubulin (Fuji Film Wako, Osaka, Japan, catalog No. 014-25041) antibodies, and anti-rabbit and anti-mouse IgG antibodies conjugated with horseradish peroxidase (GE Healthcare, Piscataway, NJ, USA) were used as primary and secondary antibodies, respectively. The proteins were detected using ImmunoStar LD (Fuji Film Wako) for MUTYH and Chemi-Lumi One Super (Nacalai Tesque) for GAPDH with an ImageQuant LAS 4000 mini image analyzer (GE Healthcare).

### Statistics

Statistical comparisons between the control and MUTYH-knockdown cells were performed using the Tukey test.

## Results

### Reduced repair of G^O^:A base pair by MUTYH knockdown

**Fig. 3:**
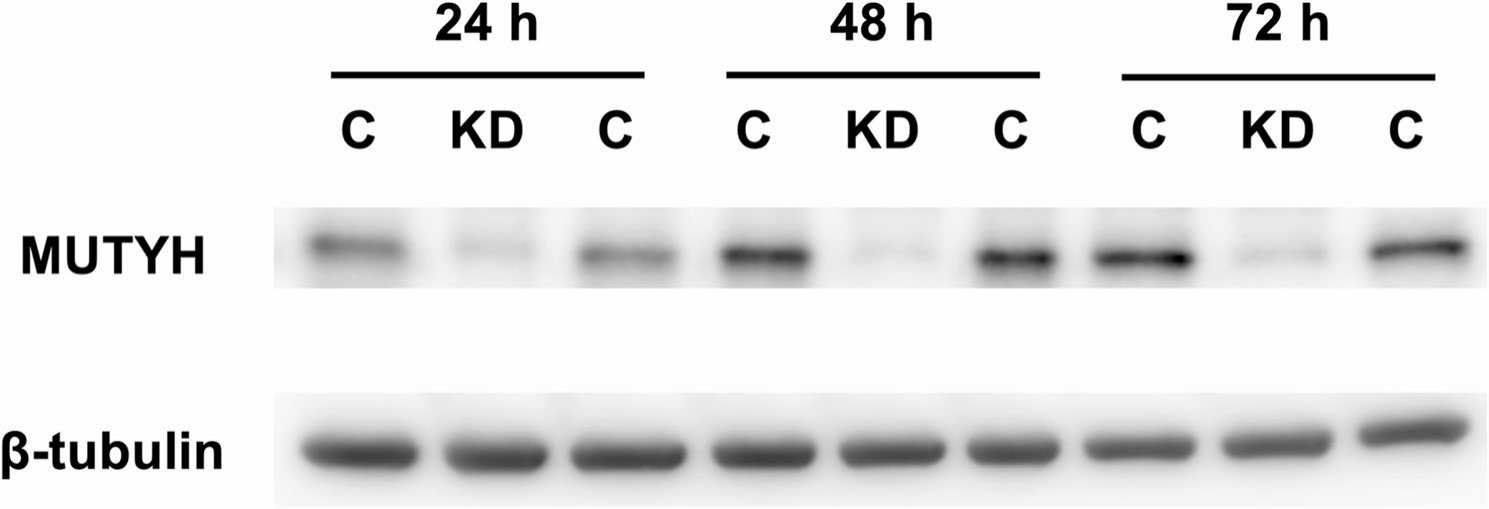
Confirmation of MUTYH knockdown by siRNA. MUTYH expression levels in U2OS cells at 24, 48, and 72 h after siRNA treatment were examined by western blot analysis. C, control cells treated with Stealth RNAi siRNA negative control; KD, knockdown cells treated with siRNA against MUTYH

We hypothesized that the excision of the undamaged A base from the G^O^:A pair by MUTYH triggers the action-at-a-distance mutations in human cells. We first reduced the MUTYH DNA glycosylase by RNA interference to examine the DNA glycosylase-driven mutations. The siRNA against MUTYH was introduced into human U2OS cells with cationic lipids and the knockdown was confirmed by western blotting. MUTYH was efficiently reduced at 24 (the time point corresponding to plasmid transfection) to 72 h (plasmid extraction) after siRNA introduction (Fig. 3). The knockdown efficiencies were 52, 75, and 72% at 24, 48, and 72 h, respectively (the means of two independent experiments).

Next, we constructed the shuttle plasmid carrying a G^O^:A pair. The *supF* gene encodes a suppressor tRNA and contains a region that is not included in the processed (matured) tRNA. This region is the pre-tRNA sequence and is editable. We altered the region to an artificial sequence, in accord with the G^O^-ODN (ODN-2, Table 1) used in our previous studies [33]. The G^O^:A pair was introduced into the pre-tRNA sequence (“position 29”) of the modified *supF* gene (Fig. 2). Plasmids containing a G:C or G:A pair were used as the controls. MUTYH reportedly excises the A base opposite G [30]. As described above, position 29 is located in the pre-tRNA region, and a small mutation at this position does not inactivate the gene. Thus, the action-at-a-distance mutations can be detected efficiently.

To confirm the reduced repair of the G^O^:A pair by the MUTYH knockdown, the sequence around position 29 of the replicated plasmid was analyzed by PCR-restriction fragment length polymorphism (RFLP) (Figure S1). The G:C-, G:A-, and G^O^:A-plasmid DNAs were transfected into U2OS cells treated with siRNA against MUTYH. The replicated DNAs were extracted from the cells at 48 h post-transfection and used as PCR templates. The region surrounding position 29 was amplified and then treated with *Bsa*HI. The PCR product with the G:C pair at position 29 is resistant to *Bsa*HI, and that with the T:A pair is digested to produce the two cleaved fragments. The ratio of the plasmids bearing the G:C and T:A base pairs was calculated by the band intensities of the intact and digested DNAs, respectively, after agarose gel electrophoresis. The PCR product derived from the G:C-plasmid was completely resistant to the enzyme, as expected. The digested-band ratio was about 50% with or without the MUTYH knockdown in the case of the G:A-plasmid (Figure S1B and C, and Table S1). This result suggested that the G:A pair, at least at this position, was a poor substrate for MUTYH. While the digested-band ratio was 26% for the G^O^:A-plasmid in the control cells, the ratio was increased to 47% in the MUTYH-knockdown cells.

Similar results were obtained using Sanger sequencing followed by quantification with the EditR program, which was developed to measure base editing efficiency (Figure S1D and E, and Table S1) [53]. These results confirmed that MUTYH was functionally suppressed in the knockdown cells.

### Increased mutant frequency by G^O^:A base pair and its suppression by MUTYH knockdown

We then transfected the G^O^:A-plasmid into the control and MUTYH-knockdown cells again, and examined the *supF* mutant frequencies of the plasmids extracted from the cells. As described in the Introduction section, MUTYH excises the unmodified A base, and not the modified G^O^ base. Thus, the single-strand break would be generated in the A-strand. This would lead to the C to U deamination in the G^O^-strand, and consequently, base-substitution mutations at the C bases of the 5'-TpC-3' sequences.

**Fig. 4:**
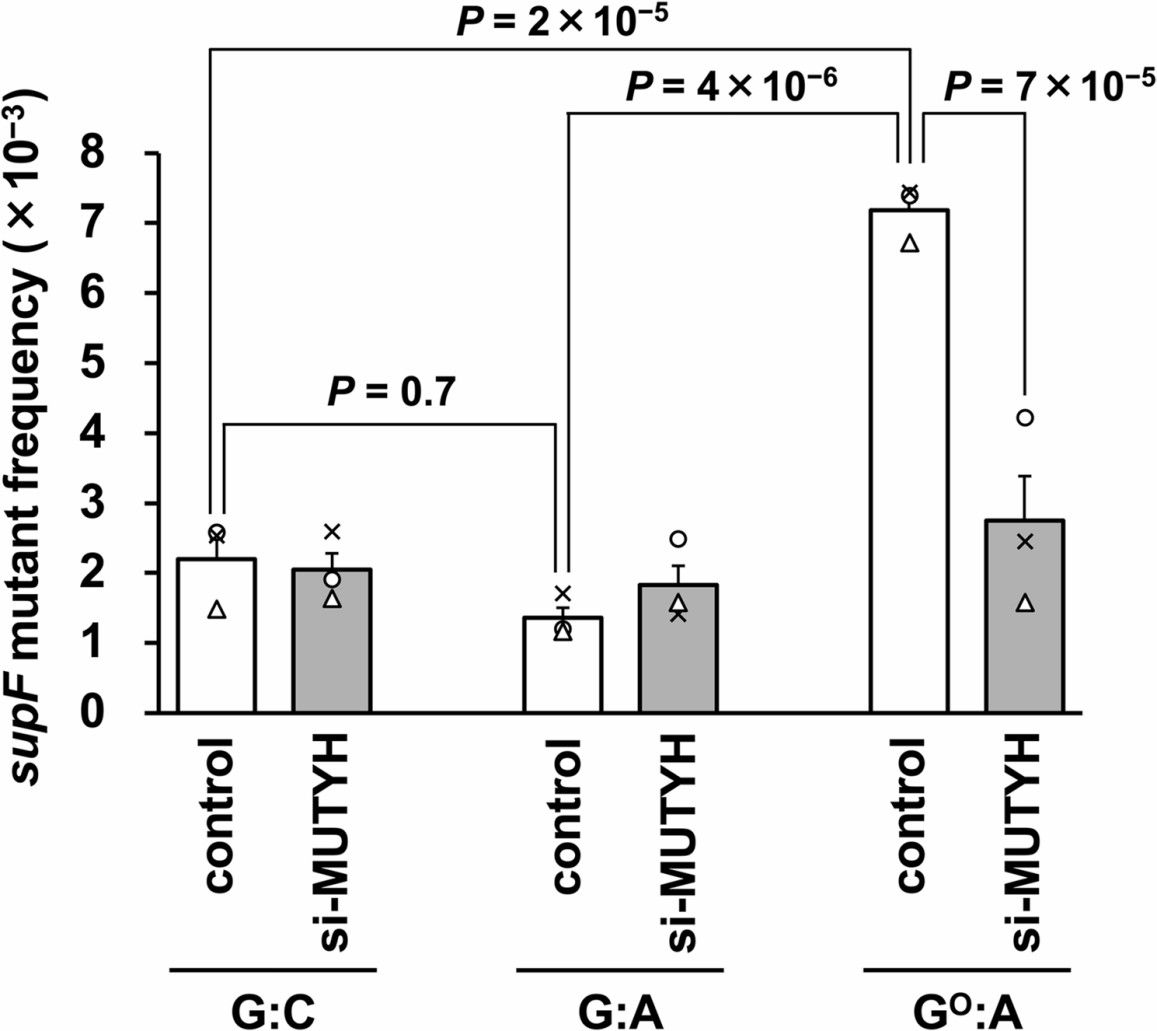
The *supF* mutant frequencies of the control G:C-, G:A-, and G^O^:A-plasmids, and the effects of the MUTYH knockdown. The transfection experiments were performed three times, and each frequency is shown as a circle, triangle, or cross. Data are expressed as the means + standard errors. Control, cells treated with Stealth RNAi siRNA negative control; si-MUTYH, cells treated with siRNA against MUTYH

The *supF* mutant frequency for the G^O^:A experimental group was 3.4-fold higher as compared to the G:C group in the control cells (Fig. 4). This result suggested that the G^O^:A pair, in addition to the G^O^:C pair [36], induced action-at-a-distance mutations. However, the *supF* mutant frequency of the G:A mispair plasmid was comparable to that of the G:C-plasmid. In contrast to our initial expectation, the G:A pair at position 29 seemed to escape from MUTYH in the cells, in agreement with the PCR-RFLP analysis results (Table S1). The *supF* mutant frequency of the G^O^:A-plasmid was decreased to a value close to that for the G:C-plasmid by the MUTYH knockdown, as expected. Thus, the mutations in the *supF* gene induced by the G^O^:A pair seemed to be driven by MUTYH. Meanwhile, the knockdown had no effect on the *supF* mutant frequency of the G:A-plasmid.

### Mutations at 5'-TpC-3' are increased by G^O^:A and decreased by MUTYH knockdown

Next, we analyzed the mutation spectra to identify the mutations contributing to the increased mutant frequency for the G^O^:A-plasmid (Tables S2-S4). Note that the mutations outside of the *supF* gene are accompanied by at least one mutation within the gene. The mutations induced by G^O^:A were detected mostly at G:C base pairs (Tables 2 and 3), and especially at the C bases in the 5'-TpC-3' contexts, the preferred substrates for APOBEC3, in the G^O^-strand (Table 4). Substitution mutations at the G bases of 5'-GpA-3' in the G^O^-strand, which was a feature of the action-at-a-distance mutations by G^O^:C, were rarely detected. Furthermore, mutations at 5'-Tp*C*-3' were reduced by the MUTYH knockdown. These results indicated that the action-at-a-distance mutations by G^O^:A occurred at the C bases of 5'-TpC-3' in the G^O^-strand. Most plasmids (> 82%) with mutations at 5'-Tp*C*-3' had the G:C pair at position 29 for the G^O^:A groups, regardless of the MUTYH knockdown.

**Table 2:** Overall mutation spectra^a^

	29G:C				29G:A				29G^O^:A			
	control		si-MUTYH		control		si-MUTYH		control		si-MUTYH	
untargeted substitution												
at A:T pair	14	(23)	6	(10)	3	(5)	5	(8)	1	(2)	1	(2)
at G:C pair	88	(147)	105	(175)	79	(132)	110	(183)	107	(178)	103	(172)
small insertion (1-2 bp)	6	(10)	1	(2)	6	(10)	4	(7)	1	(2)	4	(7)
large insertion (>2 bp)	0	(0)	0	(0)	1	(2)	2	(3)	0	(0)	0	(0)
small deletion (1-2 bp)	9	(15)	1	(2)	8	(13)	1	(2)	2	(3)	4	(7)
large deletion (>2 bp)	5	(8)	6	(10)	11	(18)	7	(12)	7	(12)	8	(13)
rearrangement or complex	5	(8)	4	(7)	8	(13)	5	(8)	1	(2)	3	(5)
unknown	7	(12)	7	(12)	6	(10)	5	(8)	4	(7)	4	(7)
total mutations	134	(223)	130	(217)	122	(203)	139	(232)	123	(205)	127	(212)
total colonies analyzed	60	(100)	60	(100)	60	(100)	60	(100)	60	(100)	60	(100)

^a^All data are represented as cases found (%)

**Table 3:** Untargeted base substitution mutations^a^

	29G:C				29G:A				29G^O^:A			
	control		si-MUTYH		control		si-MUTYH		control		si-MUTYH	
transition												
A:T → G:C	5	(11)	2	(5)	2	(5)	4	(8)	0	(0)	1	(2)
G:C → A:T	27	(60)	43	(102)	24	(55)	35	(73)	39	(76)	33	(61)
transversion												
A:T → T:A	4	(9)	3	(7)	0	(0)	1	(2)	1	(2)	0	(0)
A:T → C:G	0	(0)	0	(0)	0	(0)	0	(0)	0	(0)	0	(0)
G:C → T:A	10	(22)	14	(33)	10	(23)	24	(50)	19	(37)	26	(48)
G:C → C:G	17	(38)	17	(40)	22	(50)	12	(25)	32	(63)	19	(35)
total colonies analyzed	45	(100)	42	(100)	44	(100)	48	(100)	51	(100)	54	(100)

^a^All data are represented as cases found (%). Barcode-identical colonies are excluded

**Table 4:** Dinucleotide signatures of mutations at G and C^a^

		29G:C				29G:A				29G^O^:A			
		control		si-MUTYH		control		si-MUTYH		control		si-MUTYH	
C mutation													
	AC	1	(2)	2	(3)	1	(2)	3	(4)	0	(0)	2	(3)
	TC	35	(59)	32	(43)	31	(55)	49	(71)	80	(88)	48	(61)
	GC	1	(2)	2	(3)	0	(0)	1	(1)	0	(0)	0	(0)
	CC	3	(5)	8	(11)	5	(9)	7	(10)	8	(9)	11	(14)
total C mutations		40	(68)	44	(59)	37	(66)	60	(87)	88	(97)	61	(77)
G mutation													
	GA	14	(24)	22	(30)	18	(32)	9	(13)	2	(2)	14	(18)
	GT	2	(3)	3	(4)	0	(0)	0	(0)	0	(0)	1	(1)
	GG	2	(3)	5	(7)	1	(2)	0	(0)	1	(1)	1	(1)
	GC	1	(2)	0	(0)	0	(0)	0	(0)	0	(0)	2	(3)
total G mutations		19	(32)	30	(41)	19	(34)	9	(13)	3	(3)	18	(23)
total base substitution at G:C sites		59	(100)	74	(100)	56	(100)	69	(100)	91	(100)	79	(100)

^a^The sequence of the upper strand is shown. The percentages are shown in parentheses

**Fig. 5:**
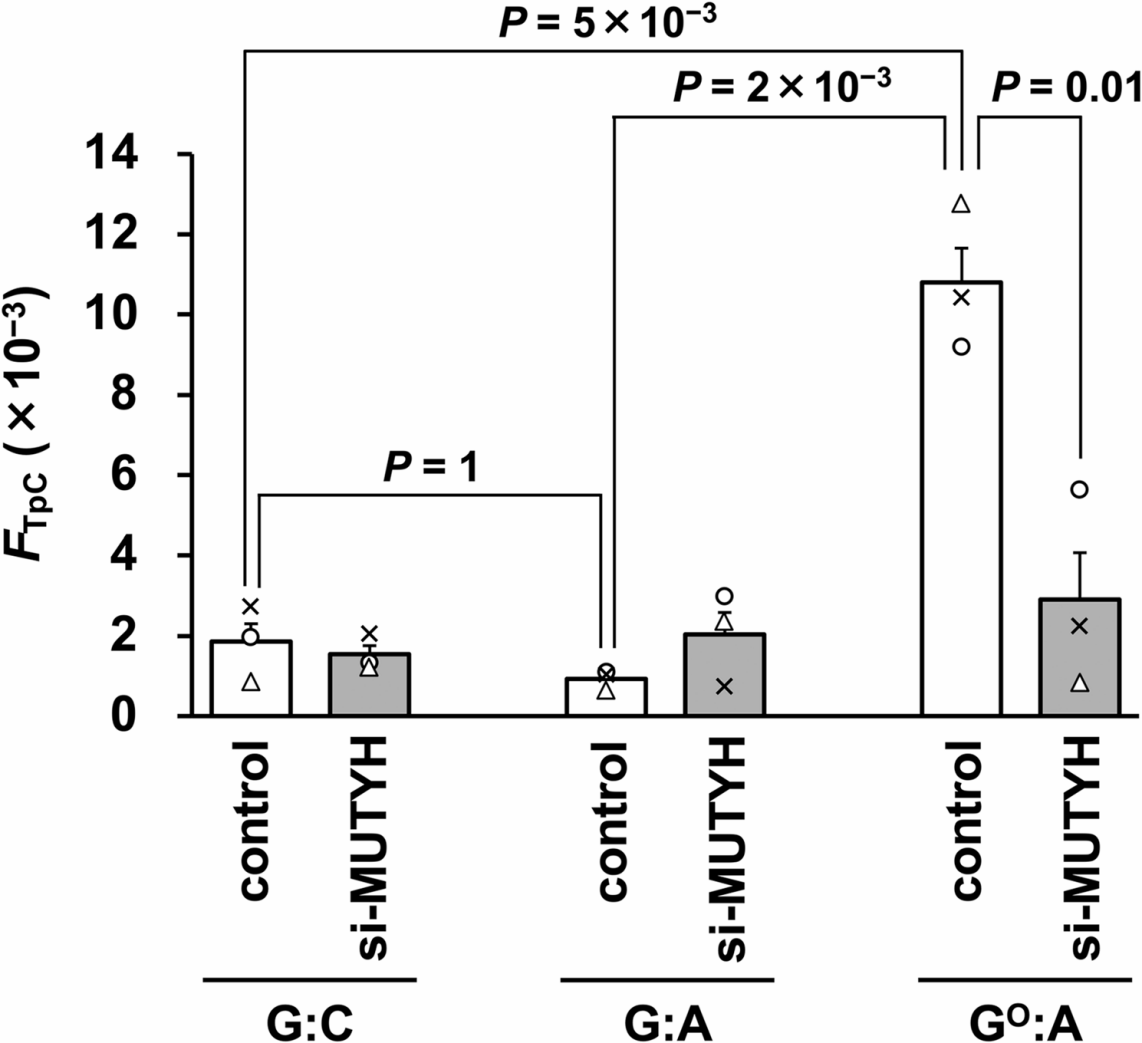
The frequencies of substitution mutations at the 5'-Tp*C*-3' dinucleotides (*F*_TpC_ value) of the G:C-, G:A-, and G^O^:A-plasmids, and the effects of the MUTYH knockdown. The transfection experiments were performed three times, and each frequency is shown as a circle, triangle, or cross. Data are expressed as the means + standard errors. Control, cells treated with Stealth RNAi siRNA negative control; si-MUTYH, cells treated with siRNA against MUTYH

The substitution mutation frequency at the C of 5'-TpC-3' (*F*_TpC_) was calculated based on the *supF* mutant frequency and mutation spectrum (Fig. 5). The *F*_TpC_ values of the G:C- and G:A-plasmids in the control cells were 1.9 × 10^–3^ and 0.9 × 10^–3^, respectively, without a significant difference. In contrast, the *F*_TpC_ value (1.1 × 10^–2^) was increased by ~ 10-fold by G^O^:A compared to G:C. In addition, the *F*_TpC_ value was reduced to 26% (2.9 × 10^–3^) by the MUTYH knockdown. Thus, these *F*_TpC_ values supported our initial hypothesis that MUTYH triggers the action-at-a-distance mutations by removing the A base paired with the G^O^ base.

## Discussion

In this study, we focused on MUTYH and the G^O^:A pair to examine whether the base excision by a DNA glycosylase potentially causes action-at-a-distance mutagenesis. We confirmed that the anticipated, untargeted mutations occurred when the G^O^:A-plasmid was transfected into U2OS cells (Figs. 4 and 5). MUTYH is expected to excise the intact A base in the G^O^:A pair. This scenario contrasts with that of the G^O^:C pair, from which OGG1 is considered to remove the damaged base (G^O^). However, in common, the subsequent steps would involve the incision of the DNA strand containing an abasic site by APE1 (see below). As a consequence of the cleavage of different strands, the 5'-Tp*C*-3' sites and the 5'-*G*pA-3' sites in the original G^O^-strand were mutated for G^O^:A and G^O^:C, respectively (Table S4) [35, 36]. Moreover, uracil, a substrate for the UNG2 glycosylase, also evokes action-at-a-distance mutations [46]. Thus, DNA glycosylases, including MUTYH, OGG1, and UNG2, cause mutations around the damaged site, although the enzymes suppress mutations at the damaged sites (*DNA glycosylase paradox*). Moreover, action-at-a-distance mutations were also elicited by nicks, an abasic site, and a ribonucleoside in DNA [46, 54, 55]. Human RNase H2 catalyzes the hydrolysis of the 5'-phosphodiester bond between 5'-DNA-RNA-3' to produce a nick [56, 57]. Therefore, the nick formation is a key step that leads to untargeted mutations.

MUTYH is a monofunctional glycosylase without lyase activity [27]. OGG1 is a bifunctional glycosylase, but its lyase activity is quite weak [23, 58]. Thus, MUTYH and OGG1 require APE1 to incise the strand containing the resultant abasic site, and the product would be the nicked DNA with 3'-hydroxyl and 5'-deoxyribose phosphate ends.

More than half of the mutants derived from the G^O^:A-plasmid replicated in the control cells had multiple base substitution mutations at 5'-Tp*C*-3' sites (Table S4). The calculated *F*_TpC_ value, the probability of a single base substitution at 5'-Tp*C*-3' sites, was 1.1 × 10^–2^ in the control cells (see above). Given that individual base substitutions occur independently and follow the Poisson distribution [59], the expected mutant frequency with two or more base substitutions would be 5.9 × 10^–5^. However, the observed mutant frequency with multiple base substitutions was 3.2 × 10^–3^, which extremely exceeds the expected frequency. These results indicate that these multiple mutations were induced simultaneously in a single event.

In contrast to the G^O^:A mispair, the G:A mispair did not increase the *supF* mutant frequency and the *F*_TpC_ value (Figs. 4 and 5). In addition, the proportions of G:C and T:A pairs at position 29 were ~ 50% after the G:A-plasmid was replicated in U2OS cells (Figure S1C and E, and Table S1). These results suggested that MUTYH rarely excises the A from the G:A mispair in cultured cells, in contrast to the in vitro enzymological studies [30]. Furthermore, the G:A mispair in the closed circular plasmid would escape from mismatch repair since the repair system only works when the DNA strand is discontinuous, in addition to bearing a mispair [60]. Thus, G:A mispair alone would not induce action-at-a-distance mutations.

Template DNA is exposed as ss DNA during replication and could be attacked by APOBEC3 [61, 62]. In the G:A-plasmid group, almost all of the 5'-Tp*C*-3' and 5'-*G*pA-3' mutations were accompanied by G:C and T:A pairs at position 29, respectively, with or without the MUTYH knockdown (Table S3). Thus, most of these mutations could be due to the deamination during the first replication of the plasmids. Likewise, the mutations at 5'-Tp*C*-3' and 5'-*G*pA-3' detected in the G:C-plasmid could occur by the deamination during DNA replication. In the case of the G^O^:A-plasmid replicated in the control cells, all plasmids with 5'-Tp*C*-3' substitution(s) had G:C at position 29, except for one plasmid with a single-base deletion at this position (Table S4). This relationship agrees with our hypothesis that the 5'-Tp*C*-3' mutations are triggered by the excision of A from G^O^:A by MUTYH, since the A removal and subsequent dCTP insertion opposite G^O^ result in the G:C pair at the G^O^:A site. Regarding the G^O^:A-plasmids replicated in the MUTYH knockdown cells, all plasmids with mutation(s) in 5'-*G*pA-3' had T:A at position 29, and 82% of the plasmids with mutation(s) in 5'-Tp*C*-3' had G:C at position 29, showing a trend similar to that observed for the G:A-plasmid. The rest of the plasmids with 5'-Tp*C*-3' mutation(s) had T:A at position 29. The mutations could be induced by the deamination with or without subsequent uracil DNA glycosylase reaction (abasic site formation) during replication: the presence of T:A at position 29 means that the A base of G^O^:A was not excised because of the MUTYH knockdown, although dATP insertion opposite G^O^ also explains this relationship. These data support the assumption that the excision of A bases by MUTYH is necessary for the action-at-a-distance mutagenesis.

After excision of the A base from G^O^:A by MUTYH and subsequent dCTP insertion by repair polymerase(s), resultant G^O^:C potentially induces mutations at 5'-*G*pA-3' through OGG1-mediated G^O^ excision [44]. However, only two plasmids with G:C at position 29 carried a 5'-*G*pA-3' substitution in the control cells. Action-at-a-distance mutations at 5'-*G*pA-3' by G^O^:C were predominantly observed upstream of G^O^ [36]. Positions 5 and 10 are only 5'-*G*pA-3' sites where mutations are detectable in the promoter-gene region upstream of position 29. Thus, the apparent mutation frequency would be low even if they occur.

The single knockdowns of OGG1 and NEIL1 partially reduced action-at-a-distance mutations by G^O^:C, but not to the background level [44, 45]. These mutations are additively decreased by the double knockdown. These observations imply that multiple DNA glycosylases, at least OGG1 and NEIL1, are involved in the removal of G^O^ from G^O^:C. In contrast, the effect of the MUTYH knockdown on the mutations induced by G^O^:A was drastic: The *supF* mutant frequency and the *F*_TpC_ value were lowered to nearly the background level (Figs. 4 and 5). MUTYH is possibly the only DNA glycosylase specific for the G^O^:A repair, since no backup enzymes for MUTYH have been reported.

Mutations at 5'-T*C*C-3' (*e.g.*, positions 114, 118, and 144) were less frequent than those at 5'-T*C*A-3', 5'-T*C*T-3', and 5'-T*C*G-3' for the G^O^:A-experimental group, consistent with the APOBEC signature (Table S5) [38, 39, 63]. In this study, no clear hotspots were observed within 5'-TpC-3' sites in the *supF* gene, although mutations at position 79 were frequently detected (Figure S2). This contrasts with our previous study, where the G^O^:C pair caused mutational hotspots at positions 5, 91, and 126 [41]. Again, note that different DNA strands would be incised in the cases of the G^O^:A and G^O^:C pairs, and that subsequently, different ss strands are hypothesized to be attacked by APOBEC3. Under this assumption, the secondary structure(s) of the ss upper strand should be important. One of the calculated secondary structures is shown in Figure S3 [64]. In this predicted structure, only positions 79, 92, and 137 are located in small loops. In cells, ss DNA binding proteins, such as RPA, could alter the structure, resulting in deamination by ABOBEC3 at different positions. The exposed ss regions might explain the distribution of the 5'-Tp*C*-3' mutations observed in this study.

The G^O^:A pair is formed by either dATP insertion opposite G^O^ or dG^O^TP incorporation opposite A by DNA pols. When the plasmid bearing a G^O^:C pair is transfected into human cells, the increase in the *F*_TpC_ value is not observed, suggesting that the contribution of the MUTYH activity toward the G^O^:A pair is negligible (Figure S4). Thus, the misincorporation of dG^O^TP would cause the action-at-a-distance mutations via the G^O^:A pair formation. MutT and MTH1 catalyze the hydrolysis of dG^O^TP and prevent its incorporation and mutation induction [47, 65–67]. The results observed in this study highlight the roles of the nucleotide pool sanitization enzymes again, as the incorporation of dG^O^TP opposite A can lead to A:T→C:G mutations at the incorporation sites and untargeted mutations.

## Conclusion

The G^O^:A pair induced the action-at-a-distance mutations via MUTYH-mediated A base removal. Our findings suggest that repair by the G^O^ system, mediated by two DNA glycosylases, OGG1 and MUTYH, could contribute to the APOBEC signatures (SBS2 and 13), in contrast to their suppressive roles against the ROS signature (SBS18).

## Supplementary Information

Supplementary Material 1.

Supplementary Material 2. 

## Data Availability

The datasets generated and/or analyzed during the current study are available from the corresponding authors on reasonable request.

## Abbreviations

BER: base excision repair
dG^O^TP: 8-oxo-7,8-dihydro-2'-deoxyguanosine 5'-triphosphate
ds: double-stranded
G^O^: 8-oxo-7,8-dihydroguanine
ODN: oligodeoxyribonucleotide
pol: polymerase
RFLP: restriction fragment length polymorphism
ROS: reactive oxygen species
SBS: single-base substitution
ss: single-stranded
