# Supplementary material for: Action-at-a-distance mutations by 8-oxo-7,8-dihydroguanine: adenine pair triggered by MUTYH
Source: Genes Environ. 2025 Oct 16;47:18. doi: 10.1186/s41021-025-00340-0 (PMC12529804; doi:10.1186/s41021-025-00340-0)
Supplement: Supplementary file 1 — Supplementary Material 1. [file 41021_2025_340_MOESM1_ESM.pdf]

Table S1. Percentages of G/T base at position 29

ImageJ		29G:C		29G:A		$29G^O$:A	
		control	si-MUTYH	control	si-MUTYH	control	si-MUTYH
experiment 1	G:C (%)	100	100	49	46	81	58
	T:A (%)	0	0	51	54	19	42
experiment 2	G:C (%)	100	99	46	46	84	57
	T:A (%)	0	1	54	54	16	43
experiment 3	G:C (%)	100	100	47	50	72	52
	T:A (%)	0	0	53	50	28	48
mean	G:C (%)	100	100	47	47	79	56
	T:A (%)	0	0	53	53	21	44
**EditR[a]**		**29G:C**		**29G:A**		**$29G^O$:A**	
		**control**	**si-MUTYH**	**control**	**si-MUTYH**	**control**	**si-MUTYH**
experiment 1	G:C (%)	99	100	46	45	78	60
	T:A (%)	3	1	55	55	22	43
experiment 2	G:C (%)	98	98	46	45	82	55
	T:A (%)	1	2	55	57	19	47
experiment 3	G:C (%)	99	100	45	45	63	48
	T:A (%)	1	1	57	57	38	53
mean	G:C (%)	99	100	46	45	74	54
	T:A (%)	2	1	56	56	26	47

[a]The ratios of the plasmids with G:C and T:A at position 29 were calculated from the respective calibration curves for G:C and T:A, and some G:C + T:A percentages exceed 100.

Table S2. Mutations detected in the *supF* gene (29G:C-plasmid)[a]

control			si-MUTYH		
-106 C → G, 101 C → T	3	(1)	-178 C → T, -166 C → A, -27 C → T, -12 C → G, 54 C → T, 55 C → T	1	(1)
-71 C → T, -29 ΔC, -27 ΔC, 79 C → G	1	(1)	-121 C → A, 101 C → G	1	(1)
-27 C → T, 101 C → G, 137 C → T	2	(1)	-12 C → A	1	(1)
-1 C → T, 23 C → T, Δ54 - 55 (+A), 85 C → A, 109 C → A	2	(1)	5 G → A	1	(1)
5 G → C	3	(3)	5 G → A, 146 G → A	1	(1)
5 G → C, 162 G → A	1	(1)	5 G → C	3	(2)
8 A → G	1	(1)	5 G → C, 20 G → A	1	(1)
11 A → G	2	(1)	5 G → C, 20 G → A, 75 G → A, 158 G → A, 200 G → A	1	(1)
12 T → C	2	(1)	5 G → C, 27 C → A	1	(1)
54 C → G	1	(1)	5 G → C, 50 G → C, 165 G → A	5	(1)
54 C → T, 55 C → A, 92 C → T	1	(1)	8 A → G	2	(1)
55 C → A, 95 C → T	1	(1)	20 G → A, 75 G → A	1	(1)
68 G → A, 75 G → T	3	(1)	41 C → A, 54 C → T, 55 C → T, 64 C → T, 137 C → T, 144 C → T	1	(1)
69 G → A	1	(1)	45 G → A, 48 G → A, 57 G → A	1	(1)
75 G → A	1	(1)	54 C → T, 55 C → A	2	(1)
79 C → G, 95 C → G	1	(1)	54 C → T, 95 C → A, 109 C → G, 137 C → T, 144 C → T	2	(1)
79 C → G, 137 C → T, 144 C → T	1	(1)	54 - 56 ΔC, 79 C → T, 199 C → T	1	(1)
79 C → T, 95 C → T, 180 C → T	2	(1)	55 C → A	2	(1)
85 C → A	5	(4)	75 G → C, 199 C → T	2	(1)
85 C → G	2	(1)	79 C → A	2	(2)
101 C → T	1	(1)	92 C → T, 95 C → T, 109 C → A	1	(1)
110 G → C	1	(1)	95 C → A, 101 C → T	1	(1)
110 G → C, 146 G → A, 158 G → C	1	(1)	95 C → G, 109 C → A, 114 C → T, 115 C → T, 144 C → T	1	(1)
114 C → A, 131 C → T, 137 C → T, 216 C → T, 221 C → T, 274 ΔC, 276 G → A	3	(1)	101 C → G	1	(1)
-3 G → A, 3 T → A, 5 G → C, 7 T → A, 12 T → C	1	(1)	101 C → T, 109 C → T	2	(1)
-3 G → A, 3 T → A, 5 G → C, 7 T → A, 12 T → C, Δ13 - 118 (+339 bp)	2	(1)	105 G → T, 146 G → A, 164 G → C	3	(1)
Δ-222 - 152	1	(1)	106 G → A	1	(1)
Δ-165 - 193 (+TCTT)	1	(1)	-3 G → A, 3 T → A, 5 G → C, Δ6 - 193 (+TATGACGATC)	1	(1)
Δ-22 - 10	1	(1)	-3 G → A, 3 T → A, 5 G → C, 7 T → A, 12 T → C	1	(1)
Δ-22 - 28 (+1870 bp)	2	(1)	Δ-3 - 114	2	(1)
Δ-5 - 85	1	(1)	Δ-41 - 47	3	(1)
Δ18 - 129	1	(1)	Δ-97 - 65 (+913 bp)	1	(1)
Δ117 - 308	1	(1)	Δ1 - 235 (+TCATCAGAGGAATATTCCCCCAGGCACTCCTTTCAAGACT)	1	(1)
WT[b]	5	(5)	Δ56 - 115	1	(1)
WT, 200ΔG	1	(1)	Δ182 - 312 (+GTCTGG), 79 C → G	1	(1)
WT, 274ΔC	1	(1)	WT	5	(3)
			WT, 210 C → T	1	(1)
			unknown	1	(1)
total colonies analyzed	60	(45)	total colonies analyzed	60	(42)

[a]Mutations detected in single colonies are represented. The sequence of the upper strand is shown. The numbers of colonies are shown on the right side. The corrected numbers based on the barcode are shown in parentheses. Positions of the G bases of 5'-GpA-3' and C bases of 5'-TpC-3' are shown in red and blue, respectively. WT (no mutation in the *supF* gene) could have the mutation in pBR327 *ori*, as analyzed in the previous study [36].

Table S3. Mutations detected in the *supF* gene (29G:A-plasmid)[a]

control	29		si-MUTYH	29	
-223 G → A, -181 G → A, -174 G → C, 75 G → C, 96 G → A, 158 G → C	T	1 (1)	-137 C → T, 79 C → A, 92 C → A, 109 C → A, 131 C → A, 76 ^ 77 (+88 bp)	G	2 (1)
-131 T → C, 20 G → A, 70 G → A, 75 G → A	T	2 (1)	-34 C → A, 79 C → G, 95 C → G	G	2 (1)
-59 C → G, 118 - 122 ΔC, 131 C → T, 137 C → T	G	2 (1)	-27 C → T, -19 C → A, 54 C → T, 114 C → T, 115 C → A, 128 C → T, 131 C → T	G	1 (1)
-47 G → A, 5 G → C	T	1 (1)	-12 C → A	G	1 (1)
-27 C → T, 54 - 55 ΔC, 56 C → T, 137 C → G	G	2 (1)	5 G → A	T	1 (1)
5 G → A	T	1 (1)	5 G → A, 20 G → A	T	2 (1)
5 G → C	G	1 (1)	5 G → C	G	1 (1)
5 G → C, 200 G → C	T	1 (1)	11 A → G	G	2 (2)
41 C → A, 95 C → T, 109 C → T, 118 C → A, 131 C → A, 137 C → T, 144 C → A	G	1 (1)	11 A → G	T	1 (1)
54 C → G, 79 C → G	G	3 (1)	12 T → C	T	1 (1)
55 C → A	G	1 (1)	41 C → A, 79 C → G, 95 C → T, 119 C → A	G	2 (1)
58 A → G	T	1 (1)	41 C → A, 109 C → G, 131 C → T, 263 C → T	T	2 (1)
75 G → C	T	2 (2)	54 C → T, 64 C → T, 85 C → T, 101 C → T	G	1 (1)
79 C → A, 92 C → A, 137 C → T, 168 C → G	G	1 (1)	54 C → T, 79 C → A, 92 C → T	G	1 (1)
85 C → T, 101 C → T, 131 C → T	G	2 (1)	54 C → T, 79 C → T, 274 C → T, 306 C → T	G	1 (1)
95 C → A, 101 C → T	G	1 (1)	54 C → T, 85 C → A, 210 C → T	T	1 (1)
101 C → G, 131 C → G	T	1 (1)	70 G → C	T	1 (1)
102 G → A	T	1 (1)	79 C → G	G	1 (1)
109 C → G	G	1 (1)	101 C → A, 194 G → A	G	1 (1)
109 C → G, 210 C → T	G	1 (1)	101 C → T	G	1 (1)
110 G → C	T	1 (1)	109 C → A	T	1 (1)
115 C → A	G	1 (1)	109 C → A, 131 C → T, 254 C → A, 274 C → G	G	1 (1)
115 C → G	G	1 (1)	109 C → G, 128 C → A, 131 C → T, 137 C → T	G	1 (1)
Δ-83 - 111	Δ	3 (1)	109 C → T	G	1 (1)
Δ-59 - 210	Δ	1 (1)	110 G → C, 158 G → C	T	1 (1)
Δ-51 - 106 (+378 bp)	Δ	1 (1)	114 C → A	T	1 (1)
Δ-51 - 164, 119 C → T	Δ	2 (1)	114 C → T, 131 C → A, 235 C → T	G	1 (1)
Δ-51 - 199 (+GAACTTGGTTAGGTACTATCTCGCGAATGGGTACT)	Δ	1 (1)	114 C → T, 131 C → A, 235 C → T	T	8 (1)
Δ-29 - -27 (+95 bp), 79 C → G, 114 C → A	G	4 (1)	115 C → G	G	1 (1)
Δ11 - 111 (+GTGCTGCGACGC)	Δ	2 (1)	122 C → A	G	1 (1)
Δ32 - 50	T	1 (1)	124 C → A	G	1 (1)
Δ44 - 72	G	2 (1)	Δ-98 - 71, 146 ΔG, 158 G → A	Δ	1 (1)
Δ94 - 231	G	2 (2)	Δ-78 - 169	Δ	1 (1)
48 - 51 ΔGG	G	1 (1)	Δ-60 - 160 (+1722 bp)	Δ	1 (1)
118 - 122 ΔC	G	3 (1)	Δ-30 - 177 (+1967 bp)	Δ	1 (1)
107 ^ 108 (+118 bp)	G	1 (1)	Δ25 - 221 (+TGA)	Δ	1 (1)
WT, 235 C → G, 274 C → T	G	1 (1)	Δ38 - 342	G	1 (1)
WT	G	4 (4)	Δ55 - 306	T	2 (2)
WT	T	1 (1)	Δ88 - 193 (+TGCTGATTT)	G	2 (2)
			Δ110 - 235, -22 T → A, 41 C → A	G	1 (1)
			WT	G	2 (2)
			WT	T	1 (1)
			WT, Δ194 - 198	G	1 (1)
			unknown		1 (1)
total colonies analyzed		60 (44)	total colonies analyzed		60 (48)

[a]Mutations detected in single colonies are represented. The sequence of the upper strand is shown. The numbers of colonies are shown on the right side. The corrected numbers based on the barcode are shown in parentheses. Positions of the G bases of 5'-GpA-3' and C bases of 5'-TpC-3' are shown in red and blue, respectively. The column highlighted in blue or pink shows the base at position 29 and "Δ" indicates that the base at position 29 is deleted. WT (no mutation in the supF gene) could have the mutation in pBR327 ori, as analyzed in the previous study [36].

Table S4. Mutations detected in the *supF* gene ($29G^{O}$:A-plasmid)[a]

control	29			si-MUTYH	29		
-254 G → T, 101 C → T	G	1	(1)	-223 G → T, 51 G → C	T	1	(1)
-33 C → T, -27 C → G, 54 C → T, 114 C → G, 131 C → G	G	1	(1)	-166 C → T, 85 C → G, -59 ΔC (+TT)	G	1	(1)
-29 C → T, 79 C → G, 118 C → T	G	1	(1)	-109 G → A, 5 G → T	T	1	(1)
5 G → A	G	1	(1)	-94 C → G, -29 C → T, -27 C → T, 23 C → T, 79 C → T, 131 C → T	G	4	(1)
40 T → A, 41 C → T, 79 C → G	G	1	(1)	-49 C → G, -29 C → G, 55 C → A, 85 C → A	G	1	(1)
41 C → A	G	1	(1)	-27 C → A, 54 C → A, 85 C → T, 92 C → T, 144 C → G	G	1	(1)
41 C → G, 79 C → G, 137 C → T	G	1	(1)	-12 C → A	G	1	(1)
41 C → G, 79 C → G, 216 C → G	G	1	(1)	5 G → A	T	1	(1)
41 C → G, 109 C → G, 131 C → T, 137 C → G	G	1	(1)	5 G → C	T	3	(3)
54 C → G	G	1	(1)	5 G → T, 20 G → T	T	1	(1)
54 C → T, 55 C → A, 95 C → A, Δ17 - 29 (+GG)	Δ	1	(1)	54 C → T, 55 C → A	G	1	(1)
54 C → T, 79 C → T, 85 C → A, 95 C → A, 109 C → G, 131 C → T	G	1	(1)	54 C → T, 85 C → T	G	1	(1)
55 C → A	G	1	(1)	55 C → A	G	2	(2)
55 C → A, 92 C → T, 95 C → T, 109 C → T	G	1	(1)	58 A → G	T	1	(1)
55 C → A, 216 C → T	G	1	(1)	62 G → T, 109 C → G, 131 C → T, 199 C → T	G	1	(1)
55 C → A, 263 C → G	G	1	(1)	79 C → G	T	1	(1)
70 G → C	G	1	(1)	79 C → G, 101 C → T, 137 C → T	G	1	(1)
79 C → A, 95 C → A, 137 C → A	G	1	(1)	79 C → G, 274 C → A	T	1	(1)
79 C → A, 109 C → G, 137 C → T, 156 C → T	G	4	(1)	79 C → T	G	1	(1)
79 C → G	G	2	(2)	85 C → A	G	2	(2)
79 C → G, 101 C → G, 274 C → G	G	1	(1)	87 G → A, 179 ΔA, 232 G → C, 240 G → C	T	2	(1)
79 C → G, 131 C → G	G	1	(1)	92 C → T, 131 C → G, 144 C → A, 216 C → T	G	1	(1)
79 C → G, 131 C → T	G	1	(1)	95 C → T, 101 C → A, 171 - 172 ΔG	G	1	(1)
79 C → G, 274 C → T	G	1	(1)	95 C → T, 101 C → T, 131 C → T	G	1	(1)
79 C → T	G	1	(1)	101 C → A, 128 C → T, 137 C → T	T	2	(1)
79 C → T, 92 C → T	G	1	(1)	101 C → A	T	1	(1)
85 C → A	G	1	(1)	101 C → T, 131 C → T	G	1	(1)
85 C → A, 95 C → G	G	1	(1)	105 G → T	G	1	(1)
85 C → A, 137 C → T	G	1	(1)	109 C → G	G	1	(1)
85 C → T	G	1	(1)	110 G → C	T	2	(2)
92 C → T, 101 C → T	G	1	(1)	110 G → T	T	1	(1)
95 C → G	G	1	(1)	114 C → A	G	1	(1)
95 C → T, 109 C → T, 119 C → T	G	1	(1)	115 C → A, 199 C → T	G	1	(1)
101 C → A	G	1	(1)	120 C → A	G	2	(2)
101 C → A, 131 C → T, 199 C → T	G	1	(1)	Δ6 - 176	Δ	1	(1)
101 C → G	G	1	(1)	Δ33 - 174	G	1	(1)
101 C → T	G	2	(1)	Δ37 - 147, 199 C → T	G	1	(1)
101 C → T, 131 C → T, 137 C → T, 263 C → T, 274 ΔC	G	1	(1)	Δ45 - 175 (+72 bp)	G	1	(1)
109 C → G	G	3	(1)	Δ66 - 193 (+1656 bp)	T	2	(2)
109 C → G, 137 C → T	G	2	(1)	Δ77 - 195	T	1	(1)
Δ-33 - 160	Δ	1	(1)	Δ91 - 105	G	1	(1)
Δ35 - 110	G	1	(1)	Δ108 - 178, 198 C → T	G	2	(1)
Δ70 - 165	G	3	(1)	Δ111 - 359, 109 C → T	G	1	(1)
Δ80 - 136	G	1	(1)	unknown		4	(4)
Δ126 - 193 (+TCTC)	G	1	(1)				
118 - 122 ΔC	G	1	(1)				
WT	G	4	(4)				
total colonies analyzed		60	(51)	total colonies analyzed		60	(54)

[a]Mutations detected in single colonies are represented. The sequence of the upper strand is shown. The numbers of colonies are shown on the right side. The corrected numbers based on the barcode are shown in parentheses. Positions of the G bases of 5'-GpA-3' and C bases of 5'-TpC-3' are shown in red and blue, respectively. The column highlighted in blue or pink shows the base at position 29 and "Δ" indicates that the base at position 29 is deleted. WT (no mutation in the supF gene) could have the mutation in pBR327 ori, as analyzed in the previous study [36].

Table S5. Trinucleotide signatures of mutations at GA and TC[a]

		29G:C				29G:A				$29G^{O}$:A			
		control		si-MUTYH		control		si-MUTYH		control		si-MUTYH	
TC mutation													
	TCA	9	(15)	5	(7)	12	(21)	14	(20)	20	(22)	13	(16)
	TCT	13	(22)	6	(8)	7	(13)	14	(20)	32	(35)	15	(19)
	TCG	7	(12)	12	(16)	8	(14)	11	(16)	22	(24)	11	(14)
	TCC	6	(10)	9	(12)	4	(7)	11	(16)	6	(7)	9	(11)
total mutations		35	(59)	32	(43)	31	(55)	50	(72)	80	(88)	48	(61)
GA mutation													
	AGA	3	(5)	3	(4)	5	(9)	0	(0)	0	(0)	1	(1)
	TGA	7	(12)	11	(15)	6	(11)	5	(7)	1	(1)	7	(9)
	GGA	0	(0)	1	(1)	2	(4)	1	(1)	1	(1)	2	(3)
	CGA	4	(7)	7	(9)	5	(9)	3	(4)	0	(0)	4	(5)
total mutations		14	(24)	22	(30)	18	(32)	9	(13)	2	(2)	14	(18)
total base substitution at G:C sites		59	(100)	74	(100)	56	(100)	69	(100)	91	(100)	79	(100)

[a]The sequence of the upper strand is shown. The percentages are shown in parentheses.
