# Supplementary material for: Action-at-a-distance mutations by 8-oxo-7,8-dihydroguanine: adenine pair triggered by MUTYH
Source: Genes Environ. 2025 Oct 16;47:18. doi: 10.1186/s41021-025-00340-0 (PMC12529804; doi:10.1186/s41021-025-00340-0)
Supplement: Supplementary file 2 — Supplementary Material 2. [file 41021_2025_340_MOESM2_ESM.pdf]

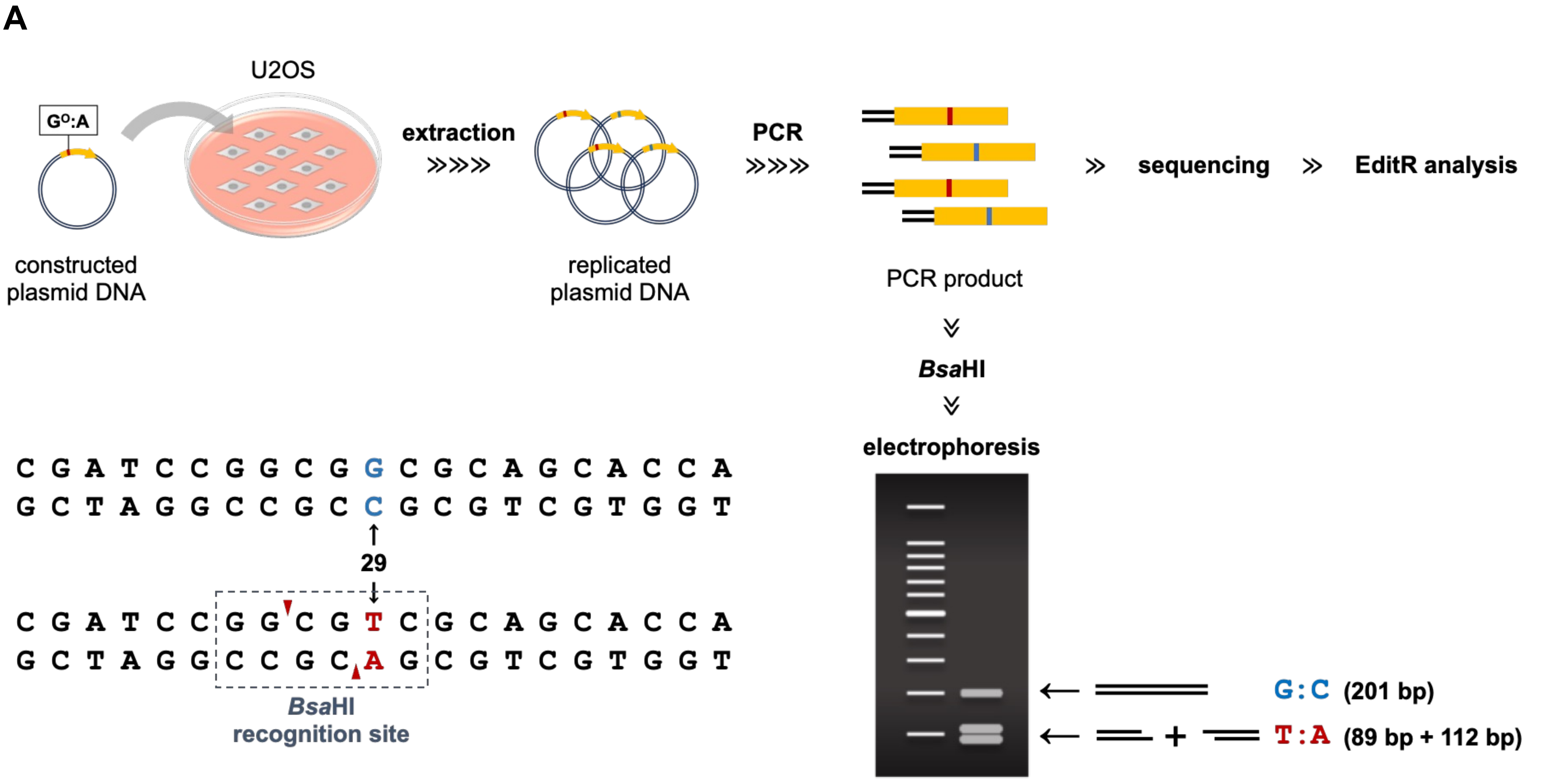
A
U2OS
G^{O}:A
constructed plasmid DNA
extraction
replicated plasmid DNA
PCR
PCR product
sequencing
EditR analysis
BsaHI
electrophoresis
C G A T C C G G C G G C G C A G C A C C A
G C T A G G C C G C C G C G T C G T G G T
29
C G A T C C G G C G T C G C A G C A C C A
G C T A G G C C G C A G C G T C G T G G T
BsaHI recognition site
G:C (201 bp)
T:A (89 bp + 112 bp)

Figure S1

B

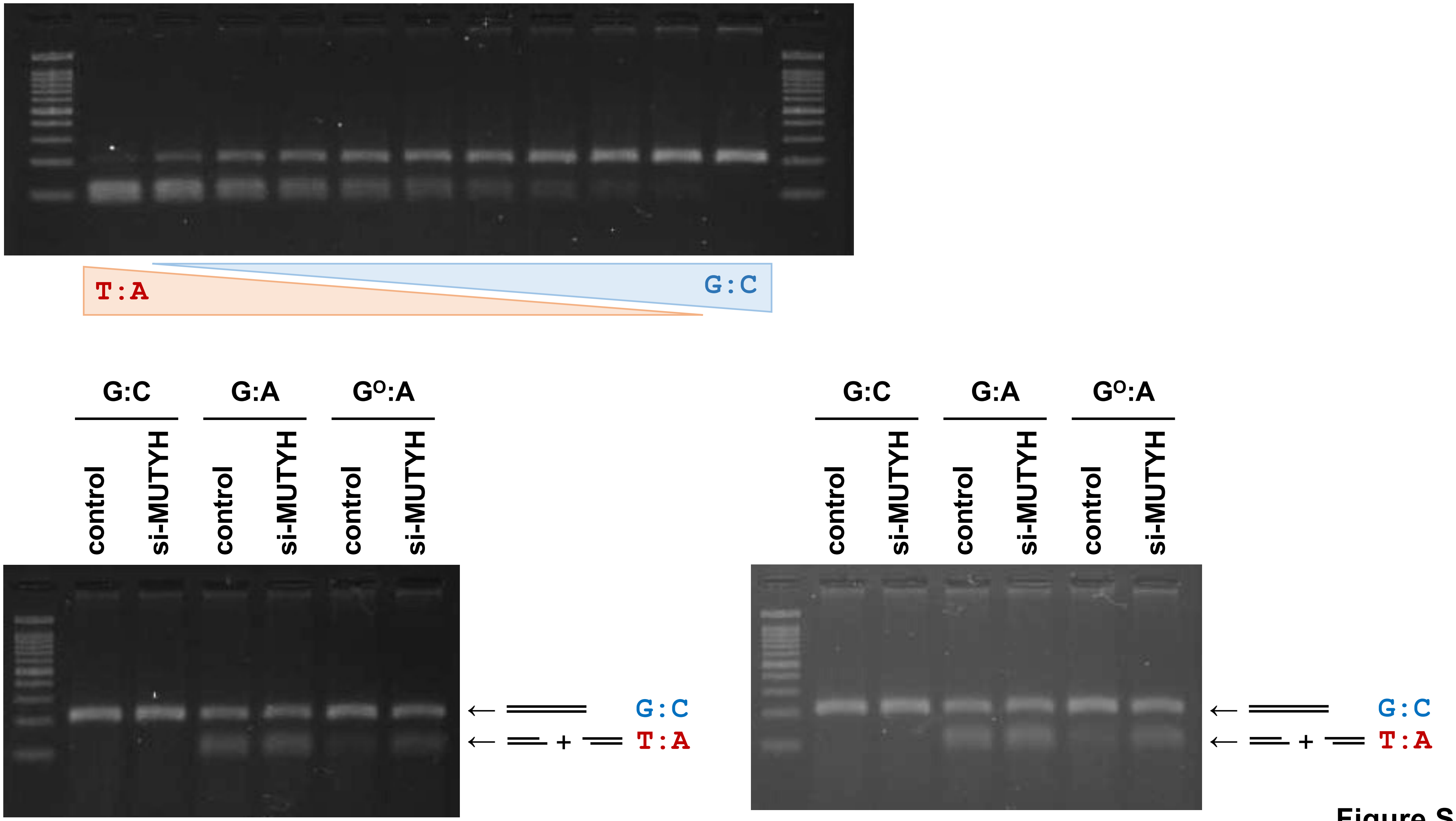
T:A
G:C
G:C
G:A
G^O:A
control
si-MUTYH
control
si-MUTYH
control
si-MUTYH
G:C
T:A
G:C
G:A
G^O:A
control
si-MUTYH
control
si-MUTYH
control
si-MUTYH
G:C
T:A

Figure S1

C

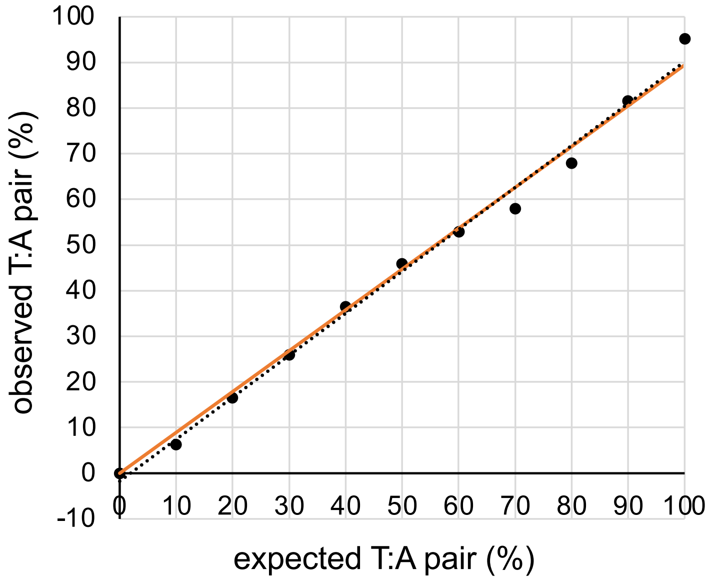

(section 0)
y = 0.8944x, R^2 = 0.9974

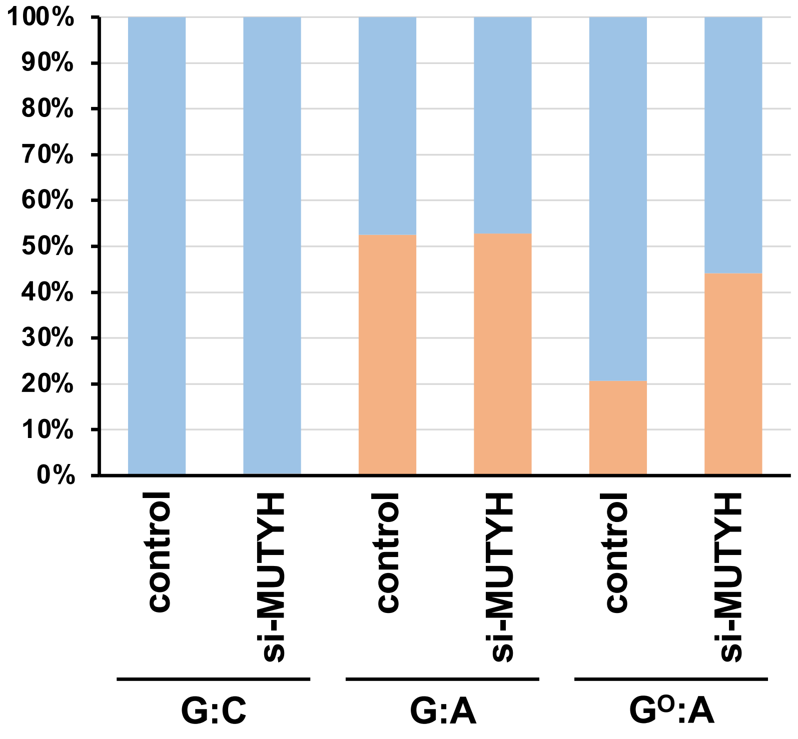

Figure S1

D

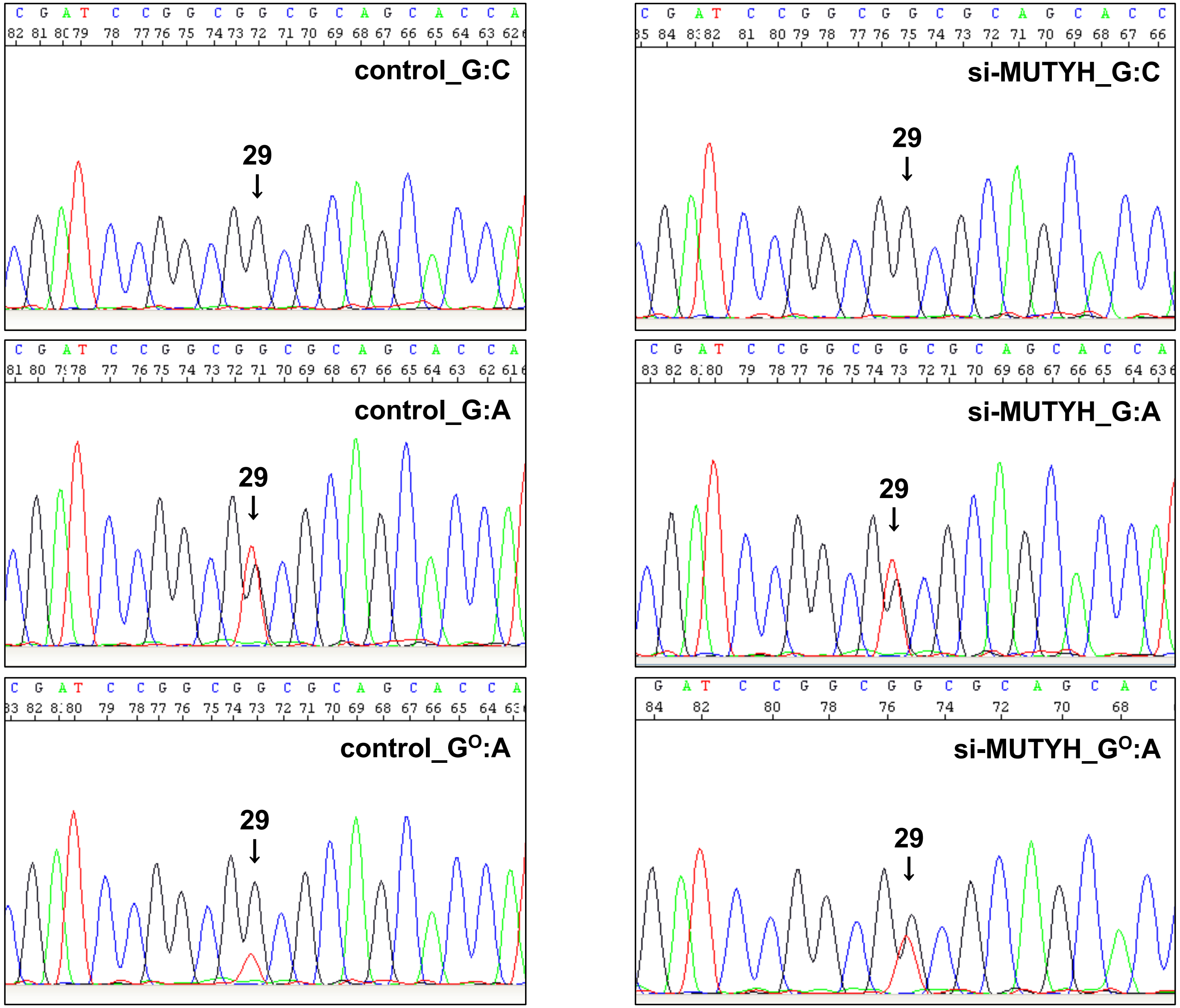
control_G:C
29
si-MUTYH_G:C
29
control_G:A
29
si-MUTYH_G:A
29
control_G^{O}:A
29
si-MUTYH_G^{O}:A
29

Figure S1

E

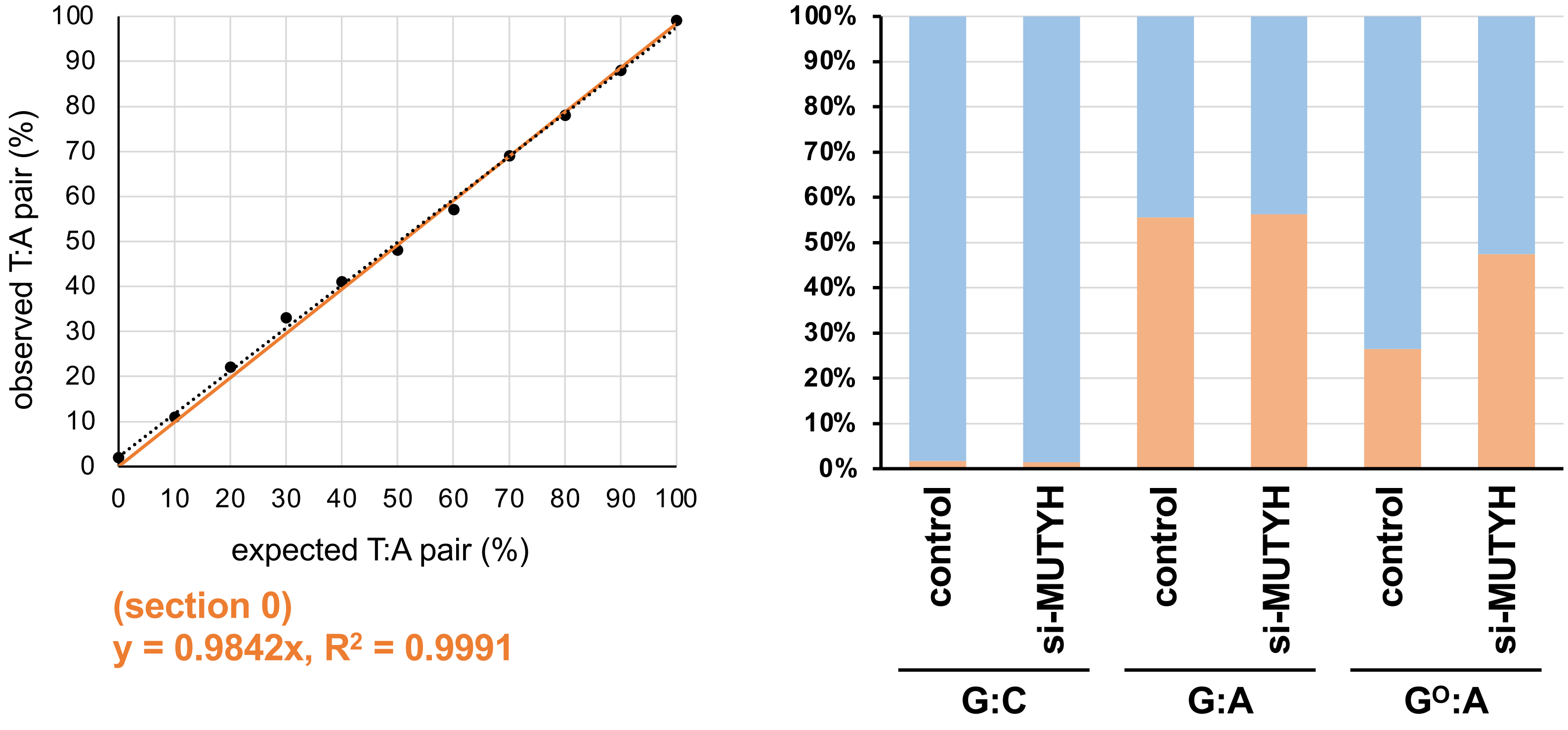

observed T:A pair (%)
expected T:A pair (%)
0
10
20
30
40
50
60
70
80
90
100
(section 0)
y = 0.9842x, R² = 0.9991
0%
10%
20%
30%
40%
50%
60%
70%
80%
90%
100%
control
si-MUTYH
control
si-MUTYH
control
si-MUTYH
G:C
G:A
G^O:A

Figure S1

Figure S1. (A) Scheme for the measurement of base pair ratios at position 29. (B) PCR-RFLP analysis of the base pair at position 29 with *Bsa*H I. The upper part shows an agarose gel image of mixtures of the G:C- and T:A-plasmids at ratios = 0:10, 1:9, 2:8, 3:7, 4:6, 5:5, 6:4, 7:3, 8:2, 9:1, and 10:0 (from left) for the calibration curve. The lower left part shows a gel image of the plasmids extracted from the transfected cells. Gels were stained with the GelRed reagent after electrophoresis. The lower right panel displays the same gel image, with the brightness adjusted linearly using the Preview software (version 11.0) to enhance band visibility. (C) The calibration curve generated from the band intensities quantified with ImageJ and the ratios of G:C- and T:A-DNAs in the extracted plasmids. The G:C and T:A ratios are indicated in blue and orange, respectively. (D) Typical electropherograms of the sequencing reaction products (corresponding to the G^{O}-strand). Black, blue, green, and red lines indicate G, C, A, and T bases, respectively. (E) The calibration curve based on the EditR analysis and the ratios of G:C and T:A of each plasmid. The peaks of A and C bases were excluded in this analysis. The G:C and T:A ratios are indicated in blue and orange, respectively. Control, cells treated with Stealth RNAi siRNA negative control; si-MUTYH, cells treated with siRNA against MUTYH.

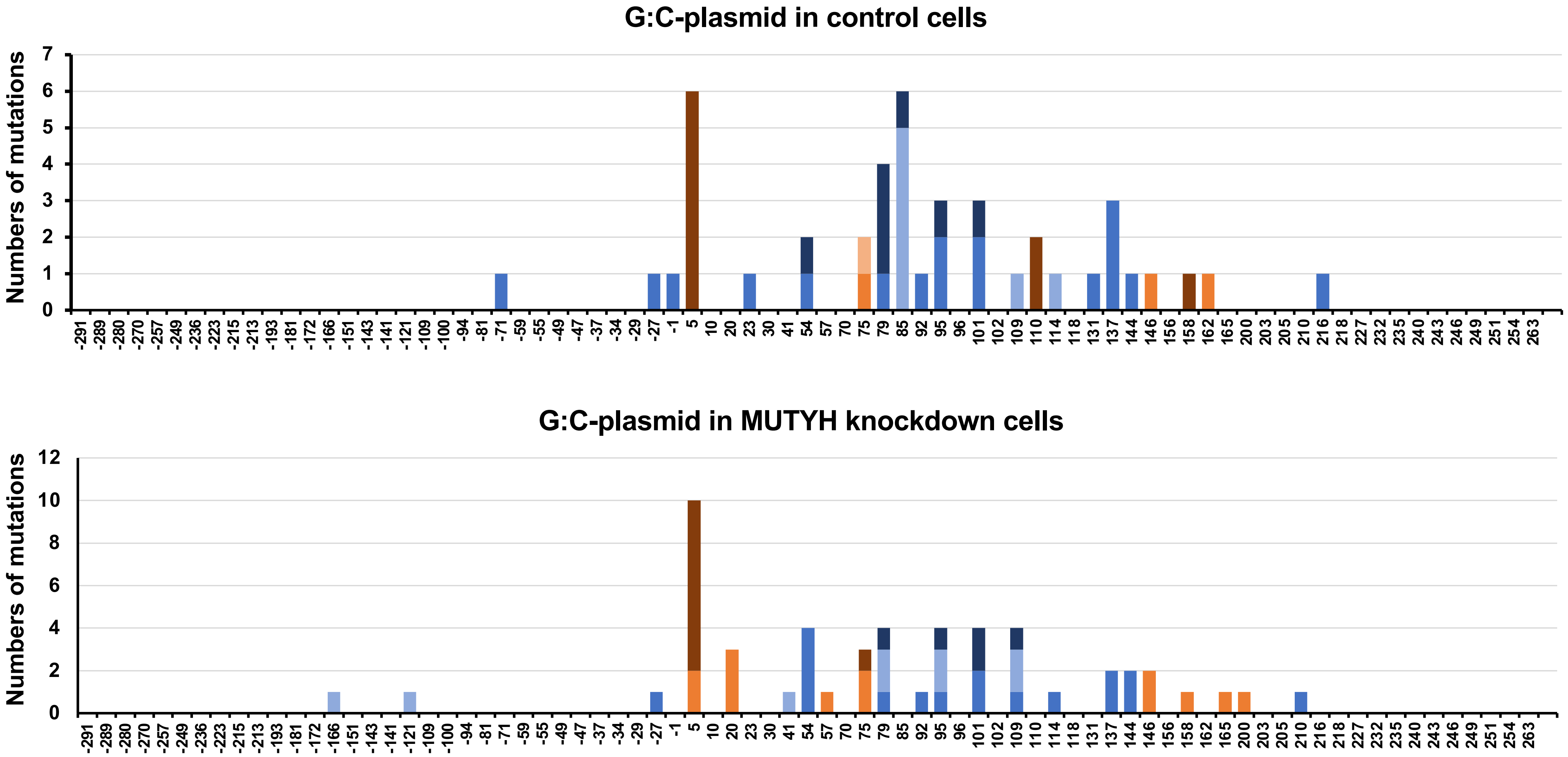
G:C-plasmid in control cells
Numbers of mutations
G:C-plasmid in MUTYH knockdown cells
Numbers of mutations

Figure S2

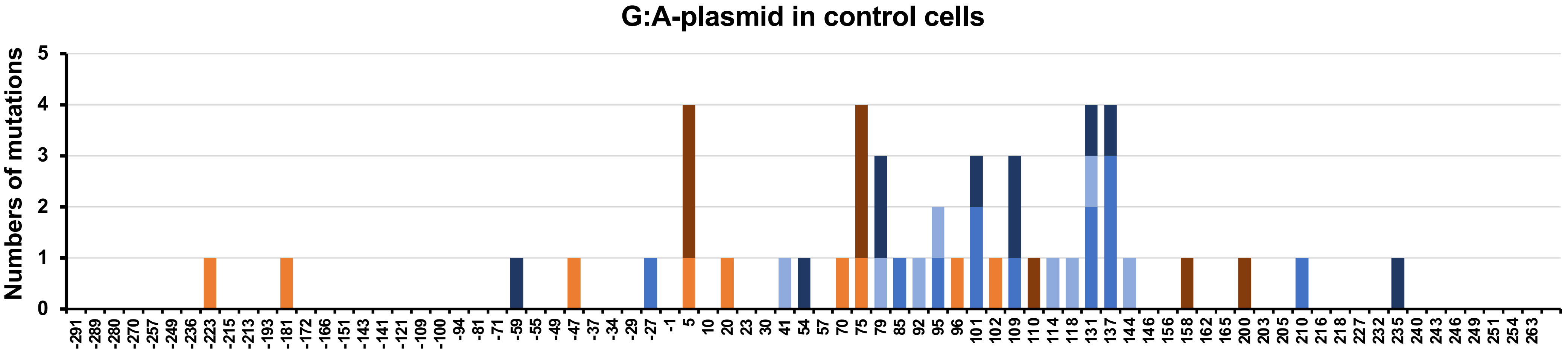
G:A-plasmid in control cells
Numbers of mutations
0
1
2
3
4
5
-291 -289 -280 -270 -257 -249 -236 -223 -215 -213 -193 -181 -172 -166 -151 -143 -141 -121 -109 -100 -94 -81 -71 -59 -55 -49 -47 -37 -34 -29 -27 -1 5 10 20 23 30 41 54 57 70 75 79 85 92 95 96 101 102 109 110 114 118 131 137 144 146 156 158 162 165 200 203 205 210 216 218 227 232 235 240 243 246 249 251 254 263

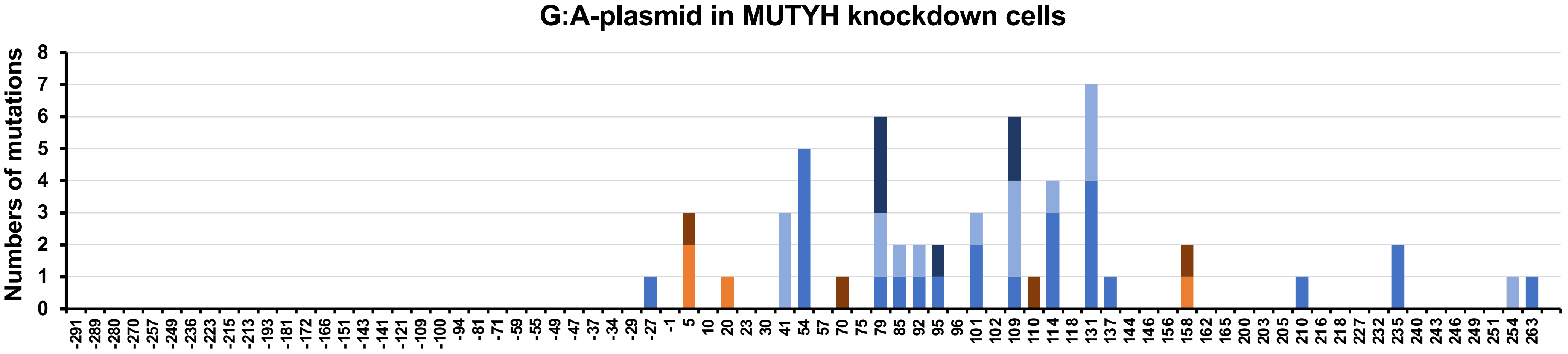
G:A-plasmid in MUTYH knockdown cells
Numbers of mutations
0
1
2
3
4
5
6
7
8
-291 -289 -280 -270 -257 -249 -236 -223 -215 -213 -193 -181 -172 -166 -151 -143 -141 -121 -109 -100 -94 -81 -71 -59 -55 -49 -47 -37 -34 -29 -27 -1 5 10 20 23 30 41 54 57 70 75 79 85 92 95 96 101 102 109 110 114 118 131 137 144 146 156 158 162 165 200 203 205 210 216 218 227 232 235 240 243 246 249 251 254 263

Figure S2

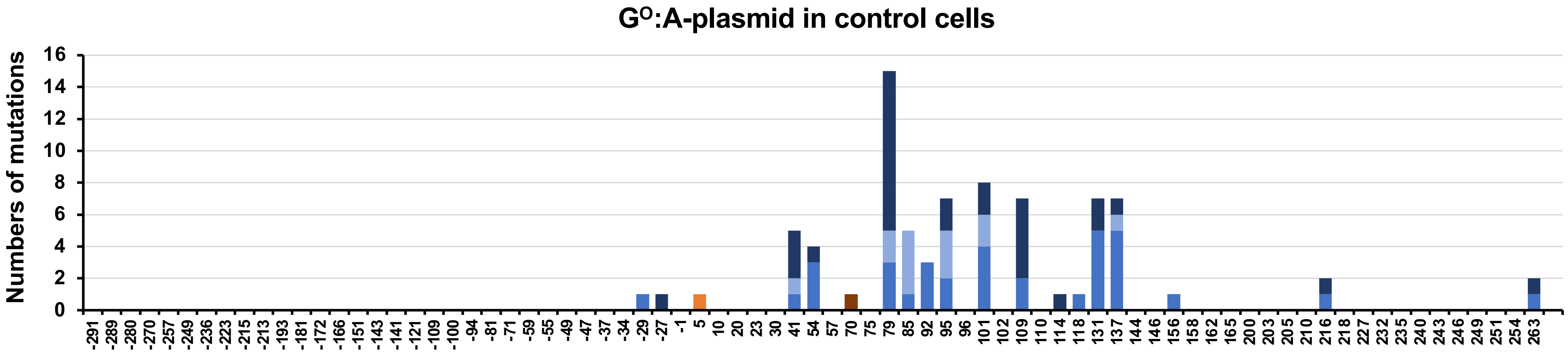

G^O:A-plasmid in control cells
Numbers of mutations
0
2
4
6
8
10
12
14
16
-291 -289 -280 -270 -257 -249 -236 -223 -215 -213 -193 -181 -172 -166 -151 -143 -141 -121 -109 -100 -94 -81 -71 -59 -55 -49 -47 -37 -34 -29 -27 -1 5 10 20 23 30 41 54 57 70 75 79 85 92 95 96 101 102 109 110 114 118 131 137 144 146 156 158 162 165 200 203 205 210 216 218 227 232 235 240 243 246 249 251 254 263

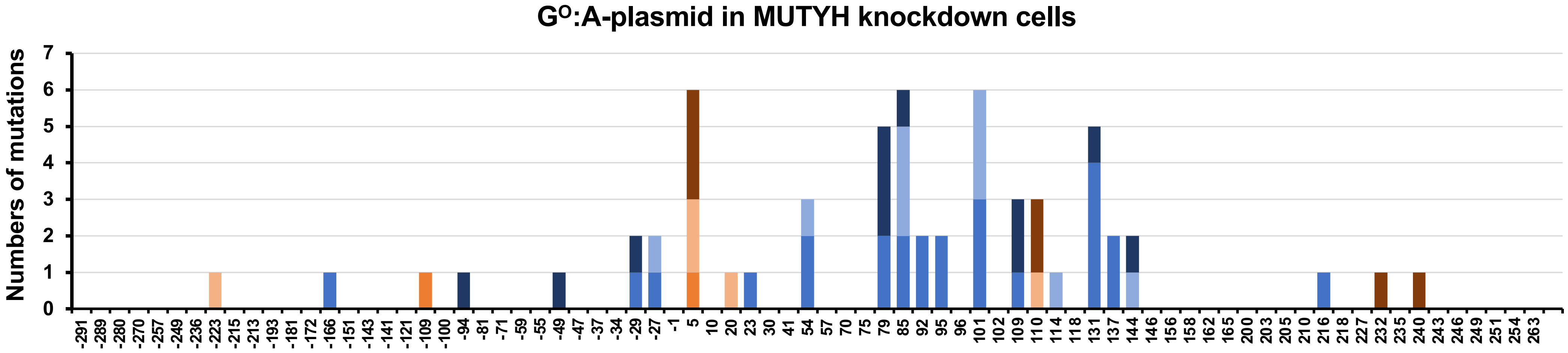

G^O:A-plasmid in MUTYH knockdown cells
Numbers of mutations
0
1
2
3
4
5
6
7
-291 -289 -280 -270 -257 -249 -236 -223 -215 -213 -193 -181 -172 -166 -151 -143 -141 -121 -109 -100 -94 -81 -71 -59 -55 -49 -47 -37 -34 -29 -27 -1 5 10 20 23 30 41 54 57 70 75 79 85 92 95 96 101 102 109 110 114 118 131 137 144 146 156 158 162 165 200 203 205 210 216 218 227 232 235 240 243 246 249 251 254 263

Figure S2

Figure S2. Distributions of the base substitutions at 5'-Tp*C*-3' and 5'-*G*pA-3'. Positions of 5'-Tp*C*-3' and 5'-*G*pA-3' within and around the *supF* gene are shown on the horizontal axis (Figure 2B). Blue, light blue, and dark blue bars indicate C→T, C→A, and C→G mutations, respectively. Orange, light orange, and dark orange bars indicate G→A, G→T, and G→C mutations, respectively.

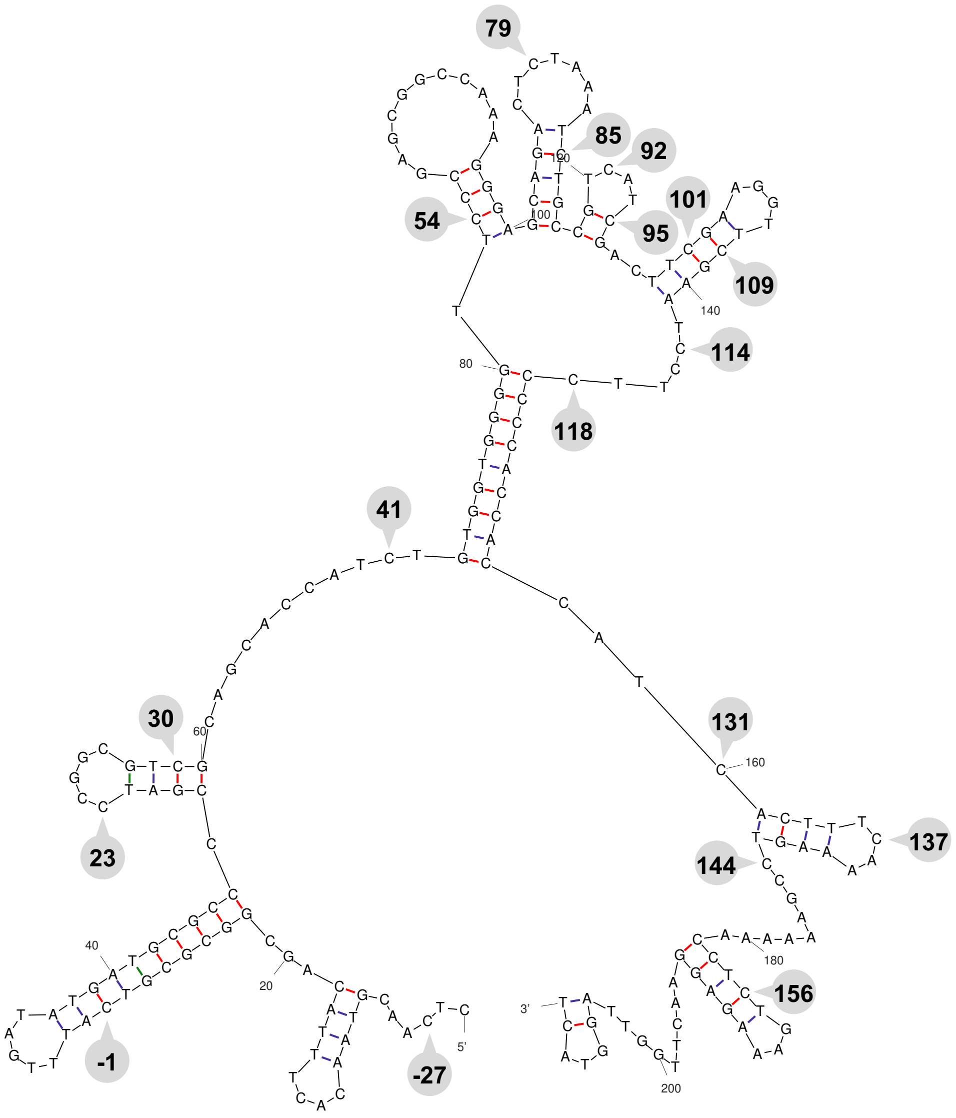

Figure S3. The predicted secondary structure with the lowest Δ*G* value, obtained by analyzing the upper strand of the plasmid DNA (from positions –29 to 181) with "DNA Folding Form" in the UNAFold web server (https://www.unafold.org/mfold/applications/dna-folding-form.php) [64].

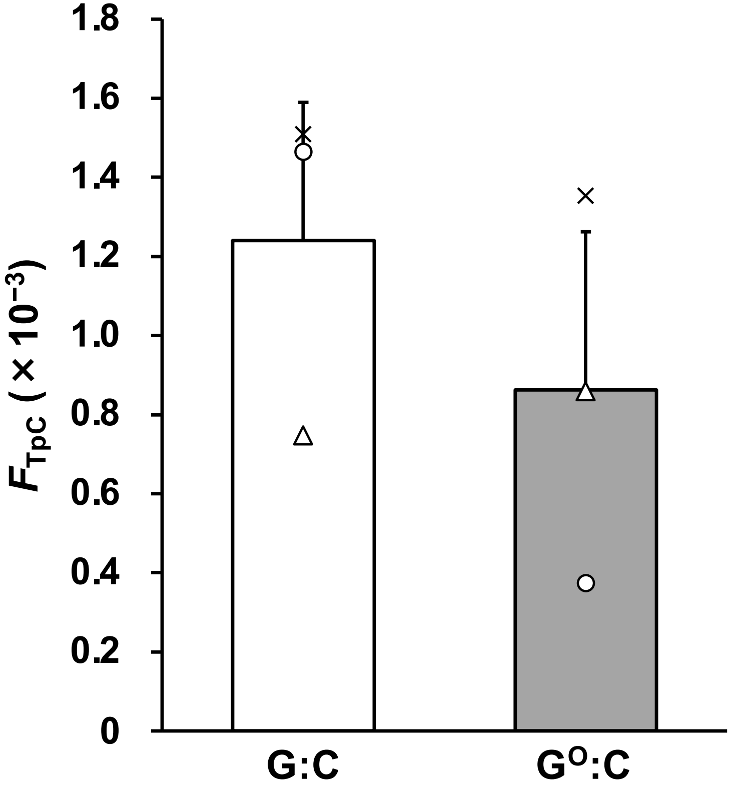

Figure S4. The frequency of substitution mutations at 5'-Tp*C*-3' dinucleotides (F_{TpC} value) in the G:C- and G^{O}:C-plasmids derived from pSB146KL-BC12(D12), based on the data obtained in a previous study [36]. The transfection experiments were performed three times, and each frequency is shown as a circle, triangle, or cross. Data are expressed as the means + standard errors.
